# Comparative and phylogenetic analyses of eleven complete chloroplast genomes of Dipterocarpoideae

**DOI:** 10.1186/s13020-021-00538-8

**Published:** 2021-11-25

**Authors:** Yang Yu, Yuwei Han, Yingmei Peng, Zunzhe Tian, Peng Zeng, Hang Zong, Tinggan Zhou, Jing Cai

**Affiliations:** 1 School of Ecology and Environment, Northwestern Polytechnical University, Xi’an, China; 2 State Key Laboratory of Quality Research in Chinese Medicine, Institute of Chinese Medical Sciences, University of Macau, 999078 Macau, China

**Keywords:** Dipterocarpoideae, Chloroplast genomes, Comparative genomics, Selected selection, Phylogenetics, DNA barcoding

## Abstract

**Background:**

In South-east Asia, Dipterocarpoideae is predominant in most mature forest communities, comprising around 20% of all trees. As large quantity and high quality wood are produced in many species, Dipterocarpoideae plants are the most important and valuable source in the timber market. The *d*-borneol is one of the essential oil components from Dipterocarpoideae (for example, *Dryobalanops aromatica* or *Dipterocarpus turbinatus*) and it is also an important traditional Chinese medicine (TCM) formulation known as “Bingpian” in Chinese, with antibacterial, analgesic and anti-inflammatory effects and can enhance anticancer efficiency.

**Methods:**

In this study, we analyzed 20 chloroplast (cp) genomes characteristics of Dipterocarpoideae, including eleven newly reported genomes and nine cp genomes previously published elsewhere, then we explored the chloroplast genomic features, inverted repeats contraction and expansion, codon usage, amino acid frequency, the repeat sequences and selective pressure analyses. At last, we constructed phylogenetic relationships of Dipterocarpoideae and found the potential barcoding loci.

**Results:**

The cp genome of this subfamily has a typical quadripartite structure and maintains a high degree of consistency among species. There were slightly more tandem repeats in cp genomes of *Dipterocarpus* and *Vatica, and* the *psbH* gene was subjected to positive selection in the common ancestor of all the 20 species of Dipterocarpoideae compared with three outgroups. Phylogenetic tree showed that genus *Shorea* was not a monophyletic group, some *Shorea* species and genus *Parashorea* are placed in one clade. In addition, the *rpoC2* gene can be used as a potential marker to achieve accurate and rapid species identification in subfamily Dipterocarpoideae.

**Conclusions:**

Dipterocarpoideae had similar cp genomic features and *psbM*, *rbcL, psbH* may function in the growth of Dipterocarpoideae. Phylogenetic analysis suggested new taxon treatment is needed for this subfamily indentification. In addition, *rpoC2* is potential to be a barcoding gene to TCM distinguish.

**Supplementary Information:**

The online version contains supplementary material available at 10.1186/s13020-021-00538-8.

## Background

Dipterocarpaceae is a small eudicot family with many giant plants, it is the symbol of South-east Asian tropical rain forests and many seasonally dry forests [1]. This family includes two subfamilies, Monotoideae and Dipterocarpoideae. Dipterocarpoideae is the larger one with 470–650 species in 13 genera [2, 3]. In South-east Asia, the dominance of Dipterocarpoideae is evident in most mature forest communities, comprising around 20% of all trees [4, 5]. Many members of this subfamily are typically 40-70 m tall, with some plants reaching as high as 85 m [6]. As large quantity and high quality wood are produced in many species of Dipterocarpoideae, they are the most important and valuable source in the timber market [7, 8]. The *d*-borneol is one of the essential oil components from Dipterocarpoideae (for example, *Dryobalanops aromatica* or *Dipterocarpus turbinatus*) [9, 10]. Borneol is also an important traditional Chinese medicine (TCM) formulation known as “Bingpian” in Chinese, with antibacterial [11], analgesic and anti-inflammatory effects [12] and can enhance anticancer efficiency [13]. Thus, borneol has been widely used in the fields of medicine, pesticide and chemical industry [14]. This TCM has been recorded in Newly Revised Canon of Materia Medica (Xinxiu Bencao) for more than 1300 years. Due to the medicinal and economic values of Dipterocarpoideae, the species have been the targets of woodcutting for long history. Some species such as *Parashorea chinensis* and *D. aromatica* even become endangered because of the over-harvesting [15, 16]. Although Dipterocapoideae is important to forest ecology, conservation and medicine, little is known about the genetics of those species. The classifications of Dipterocarpoideae have been reported before, while delineation of genus *Parashorea* and *Shorea* still remains controversial, due to the difficulty in identifying these plants leads to an uneven quality of borneol medicinal materials. Chloroplast (cp) genome information will prove essential to solve this problem. Recently, the whole cp genomes of nine species in Dipterocarpoideae were sequenced and analyzed [17, 18]. Here we sequenced, assembled and annotated the cp genomes of eleven species in four genera with the highest species richness in Dipterocarpoideae (*Hopea mollissima, Hopea odorata*, *Shorea henryana*, *Shorea roxburghii*, *Shorea leprosula*, *Dipterocarpus gracilis*, *Dipterocarpus alatus, Dipterocarpus intricatus*, *Vatica xishuangbannaensis, Vatica odorata, Vatica rassak*). Further, we performed a comprehensive evolutionary analysis of the cp genomes of 20 species from Dipterocapoideae and identified barcoding loci that could be used for species identification.

## Materials and methods

### Sample collection, DNA extraction, and sequencing

The fresh and healthy leaves of eleven species (*Hopea mollissima, Hopea odorata*, *Shorea henryana*, *Shorea roxburghii*, *Shorea leprosula*, *Dipterocarpus gracilis*, *Dipterocarpus alatus, Dipterocarpus intricatus*, *Vatica xishuangbannaensis, Vatica odorata, Vatica rassak*) were collected from the Xishuangbanna Tropical Botanical Garden, Chinese Academy of Sciences, (101°25′ E, 21°41′ N) and were immediately quick-frozen in liquid nitrogen. The total genomic DNA was extracted from leaf tissues with a modified Cetyl Trimethyl Ammonium Bromide (CTAB) method [19]. All genome DNA were sequenced with an Illumina NovaSeq 6000 platform by Biomarker Technologies, Inc (Beijing, China). The clean reads were more than 5,000 x coverage of each whole cp genome.

### Genome assembly and annotations

We used Getorganelle v1.7.1 [20] and NOVOPlasty v4.2 [21] to assemble chloroplast genome respectively, and selected the more complete result as the final genome. Five cp genomes were assembled using Getorganelle v1.7.1 (*H. mollissima*, *D. gracilis*, *D. alatus*, *D. intricatus*, *V. odorata*) and other six species cp genome using NOVOPlasty v4.2 (*H. odorata*, *S. henryana*, *S. roxburghii*, *S. leprosula*, *V. xishuangbannaensis*, *V. rassak*). The contigs were examined based on the complete chloroplast sequence of *D. turbinatus* (GenBank Accession Number: NC_046842) using the “Map to Reference” function of Genious Prime 2021.0.3 (https://www.geneious.com). We modified the relative position and direction of each contig. Then, the reads were applied to polish the assembled contigs using Nextpolish [22] to fill the gap. The newly assembled chloroplast genomes were annotated using Plastid Genome Annotator (PGA) software [23] with the cp genome of *D. turbinatus* as reference, whereas the tRNA genes were further verified by ARAGORN v1.2.38 [24] and tRNAscan-SE v2.0.7 [25], and then checked manually. Fully annotated plastomes of circular diagram were drawn by OrganellarGenomeDRAW (OGDRAW) [26].

Repeat sequences were detected using Tandem Repeats Finder (TRF) version 4.09 [27] and RepeatMasker version 1.317 (http://www.repeatmasker.org) with default parameters. The Perl script auto_repeat.pl from Zhouheling (zhouheling@genomics.org.cn) was used to analyze four types of Transposable Elements -DNA transposons, LINE (long interspersed nuclear elements), SINE (short interspersed nuclear elements) and LTR (long terminal repeats) in the chloroplast genomes of Dipterocarpoideae species.

### Comparative analyses

To investigate the divergence in the chloroplast genome, the identity across the whole complete cp genomes were visualized using the shuffle-LAGAN program of mVISTA v2.0 program [28] for the 23 species, with the *H. mollissima* genome as the reference. To detect the variation in the LSC/IR/SSC boundaries of Dipterocarpoideae chloroplast genomes, all 20 chloroplast genomes of Dipterocarpoideae species were compared by drawing in Adobe Illustrator CC2019 (https://adobe.com/products/illustrator). Codon usage in these genes was assessed using the program codonW [29]. Six values were used to estimate the extent of bias toward codons: the codon adaptation index (CAI), codon bias index (CBI), frequency of optimal codons (Fop), the effective number of codons (ENc), GC content of synonymous third codons positions (GC3s) and the relative synonymous codon usage values (RSCU).

### Species pairwise K_a_/K_s_ ratios and positive selection analysis

Pairwise K_a_/K_s_ ratios of all species were calculated using the concatenated 50 single-copy genes alignments with K_a_K_s_ Calculator [30]. Positive selections in Dipterocarpoideae were tested based on a species tree we built. PRANK v170427 [31] was used to perform multiple alignments for the protein-coding DNA sequences within each single gene. The alignments of each dataset were then fed into the Codeml program in the PAML package [32] to identify positively selected genes. Chi-square test p value < 0.05 is positive.

### Phylogenetic inference

We downloaded 12 published chloroplast genome sequences (three as the outgroup taxa) from Genbank that were included in the analyses to perform the phylogenetic reconstruction. Firstly, all single-copy genes were extracted from 23 taxa, and alignments of each gene were generated and trimmed. Secondly, these alignments were concatenated which were used for phylogenetic analysis. Finally, phylogenetic trees were constructed using Bayesian analysis (BI) methods with MrBayes v3.2.2 [33], Maximum likelihood (ML) method with PhyML v3.0 [34] and Neighbour-joining (NJ) method with TreeBeST v1.9.2 [35]. The supporting branches were assessed with 100 rapid bootstrapping replicates.

### *p*-distance calculation

To screen for rapidly evolving regions of some marker genes, we aligned the target genes by MUSCLE [36] after the annotation of 20 cp genomes of Dipterocarpoideae species. The FASTA format file was then transformed into mega format by MEGA7 [37]. Estimation of sequence divergence was expressed as the *p*-distance quantification using the Kimura 2-parameter model [38].

## Results

### Comparison among chloroplast genomic features in Dipterocarpoideae

**Fig. 1:**
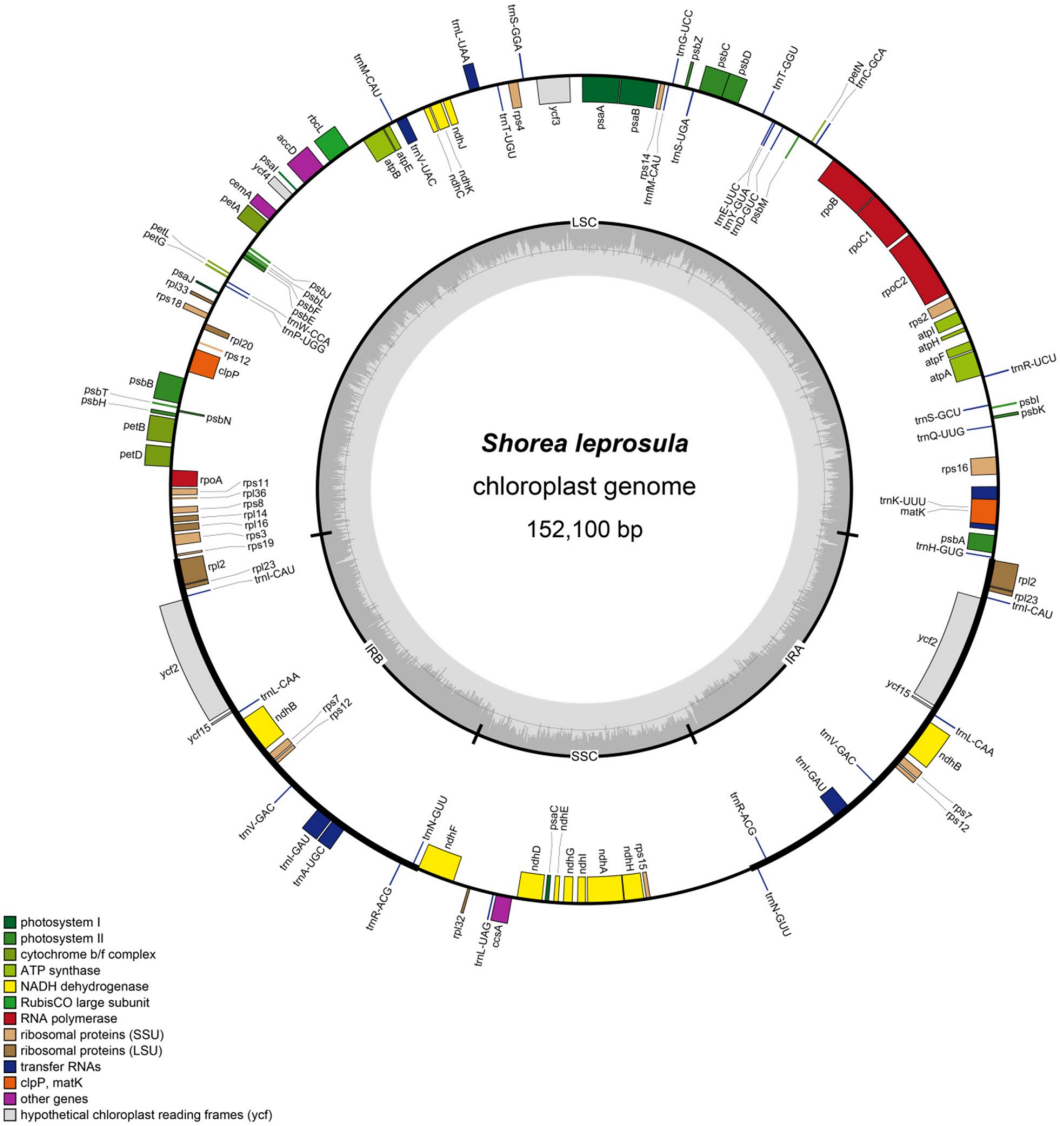
Gene map of *S. leprosula* chloroplast genomes. Genes inside the circle are transcribed clockwise, genes outside are transcribed counter-clockwise. Genes are color-coded to indicate functional groups. The dark gray area in the inner circle corresponds to guanine-cytosine (GC) content while the light gray corresponds to the adenine-thymine (AT) content of the genome. The small (SSC) and large (LSC) single-copy regions and inverted repeat (IRa and IRb) regions are noted in the inner circle

The chloroplast genome size of *V. xishuangbannaensis* (151,011 bp) was found to be the smallest and *S. leprosula* (152,100 bp) was found to be the largest (Fig. 1, Additional file 1: Fig. S1). The lengths of LSC, SSC, and IR of the 11 species are also shown in Table 1. In these species we found 110–111 unique genes including 78–79 protein coding genes, four rRNA genes, and 28 tRNA genes (Tables 1, 2).

**Table 1:** Characteristics of the chloroplast genomes of eleven Dipterocarpoideae species

Taxon	Size (bp)	LSC length (bp)	SSC length (bp)	IR length (bp)	GC conten t (%)			
					Total	LSC	SSC	IR
*D .gracilis*	152,015	83,302	20,237	24,238	37.2	35.2	31.5	43.0
*D. alatus*	151,638	83,101	20,199	24,169	37.3	35.3	31.7	43.0
*D. intricatus*	151,911	83,169	20,310	24,216	37.2	35.2	31.5	43.0
*H. mollissima*	151,497	84,281	19,702	23,757	37.4	35.3	31.9	43.3
*H. odorata*	151,745	84,529	19,678	23,769	37.3	35.3	31.9	43.3
*S. leprosula*	152,100	84,226	19,940	23,967	37.2	35.2	31.6	43.1
*S. roxburghii*	151,795	84,406	19,833	23,778	37.2	35.2	31.6	43.2
*S. henryana*	151,685	84,144	19,865	23,838	37.4	35.3	31.8	43.2
*V. xishuangbannaensis*	151,011	83,210	20,029	23,886	37.2	35.2	31.3	43.2
*V. odorata*	151,493	83,532	20,067	23,947	37.2	35.2	31.3	43.1
*V. rassak*	151,394	83,380	20,204	23,905	37.2	35.2	31.3	43.1

**Table 2:** Genes difference of the chloroplast genomes of eleven Dipterocarpoideae species

Taxon	Number of genes	Protein-coding genes	rRNA genes	tRNA genes	Genes content difference	
					*rps16*	*ycf15*
*D .gracilis*	110	78	4	28	N	Y
*D. alatus*	110	78	4	28	N	Y
*D. intricatus*	110	78	4	28	N	Y
*H. mollissima*	111	79	4	28	Y	Y
*H. odorata*	111	79	4	28	Y	Y
*S. leprosula*	111	79	4	28	Y	Y
*S. roxburghii*	111	79	4	28	Y	Y
*S. henryana*	110	78	4	28	Y	N
*V. xishuangbannaensis*	110	78	4	28	Y	N
*V. odorata*	111	79	4	28	Y	Y
*V. rassak*	111	79	4	28	Y	Y

*Y* The gene exists in this species; *N* The gene does not exist in this species

**Fig. 2:**
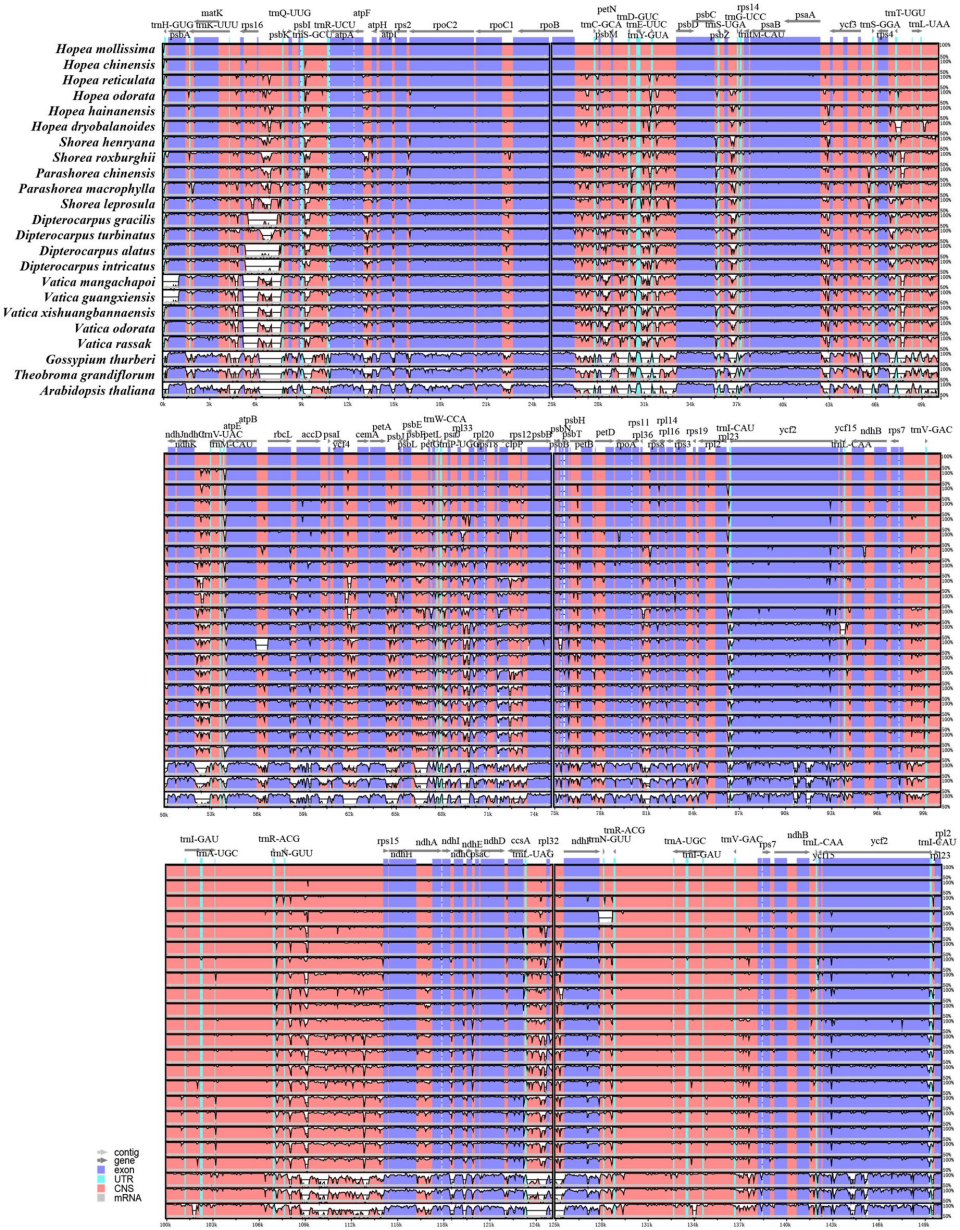
The chloroplast genomes of all 23 different species were analyzed by shuffle-LAGAN program. The percentage of identity is shown on the vertical axis, which ranges from 50–100%, while the horizontal axis represents the position in the chloroplast genome. Each arrow indicates the annotated gene in the reference genome and the direction of its transcription. Genomic regions are color-coded into exons, tRNA, conserved non-coding sequences, and mRNA

The mVISTA program was further used to align the cp genomes and visualize the pattern of sequence identity along the whole chloroplast genome of the 23 species including 20 Dipterocarpoideae species and three outgroups, using the annotation for *H. mollissima* as a reference (Fig. 2). Compared with the three outgroups, all 20 chloroplast genomes Dipterocarpoideae species displayed similar structure and gene order. The coding regions were more conserved than non-coding regions in all the species tested. In addition, LSC and SSC regions had a larger divergence than the IR regions, which has been observed in cp genome study of other taxa [39, 40]. In total, all 20 Dipterocarpoideae species showed conserved gene and gene organization.

**Fig. 3:**
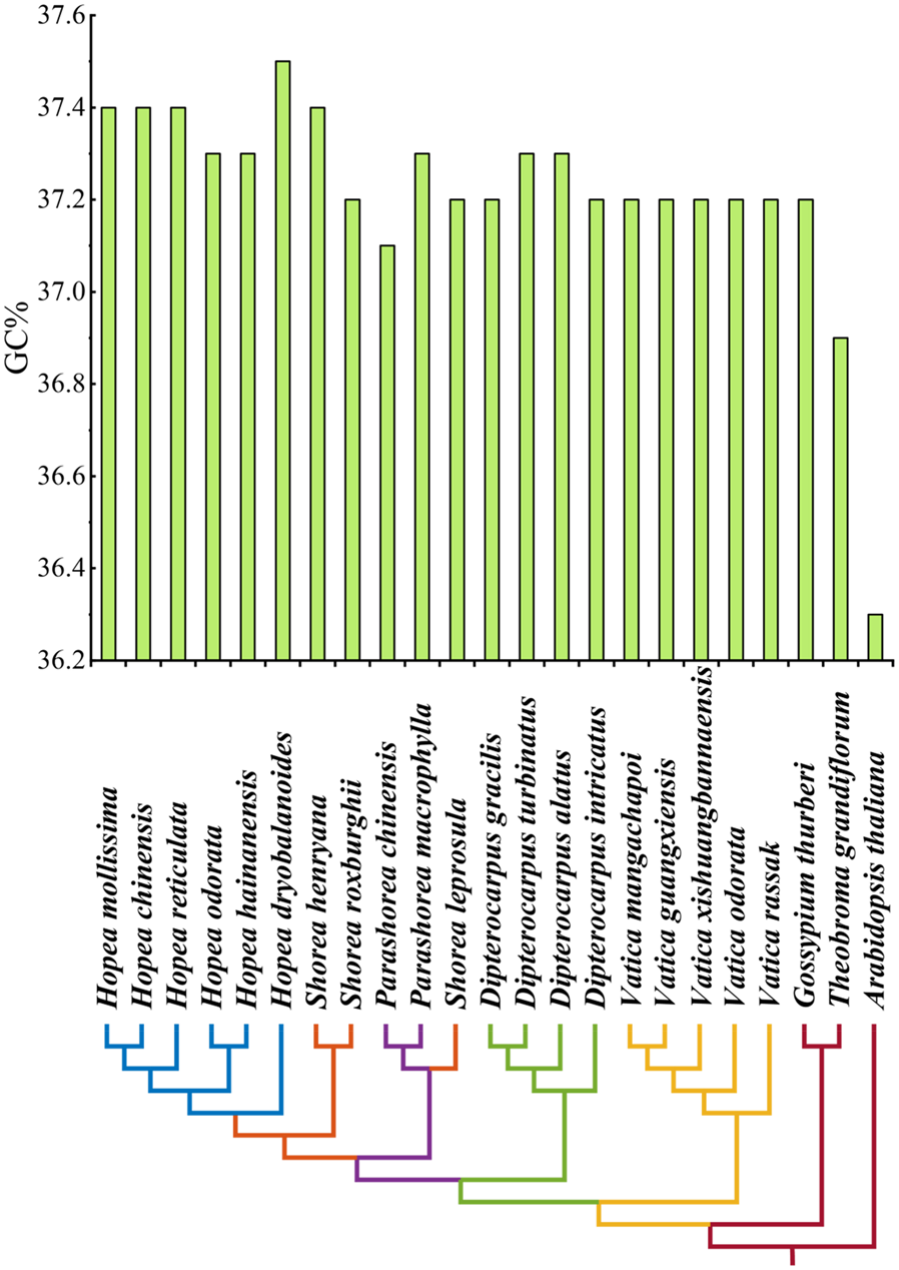
Changes in chloroplast GC content of all 23 species

The overall guanine-cytosine (GC) content was also very conserved, ranged only from 37.1% (*P. chinensis*) to 37.5% (*H. dryobalanoides*) in Dipterocarpoideae. GC content in the LSC, SSC and IR regions was 35.2–35.3%, 31.3–31.9% and 43.0–43.2%, respectively. IR regions showed high GC content compared to the LSC and SSC regions (Table 1; Fig. 3).

### Contraction and expansion of inverted repeats

**Fig. 4:**
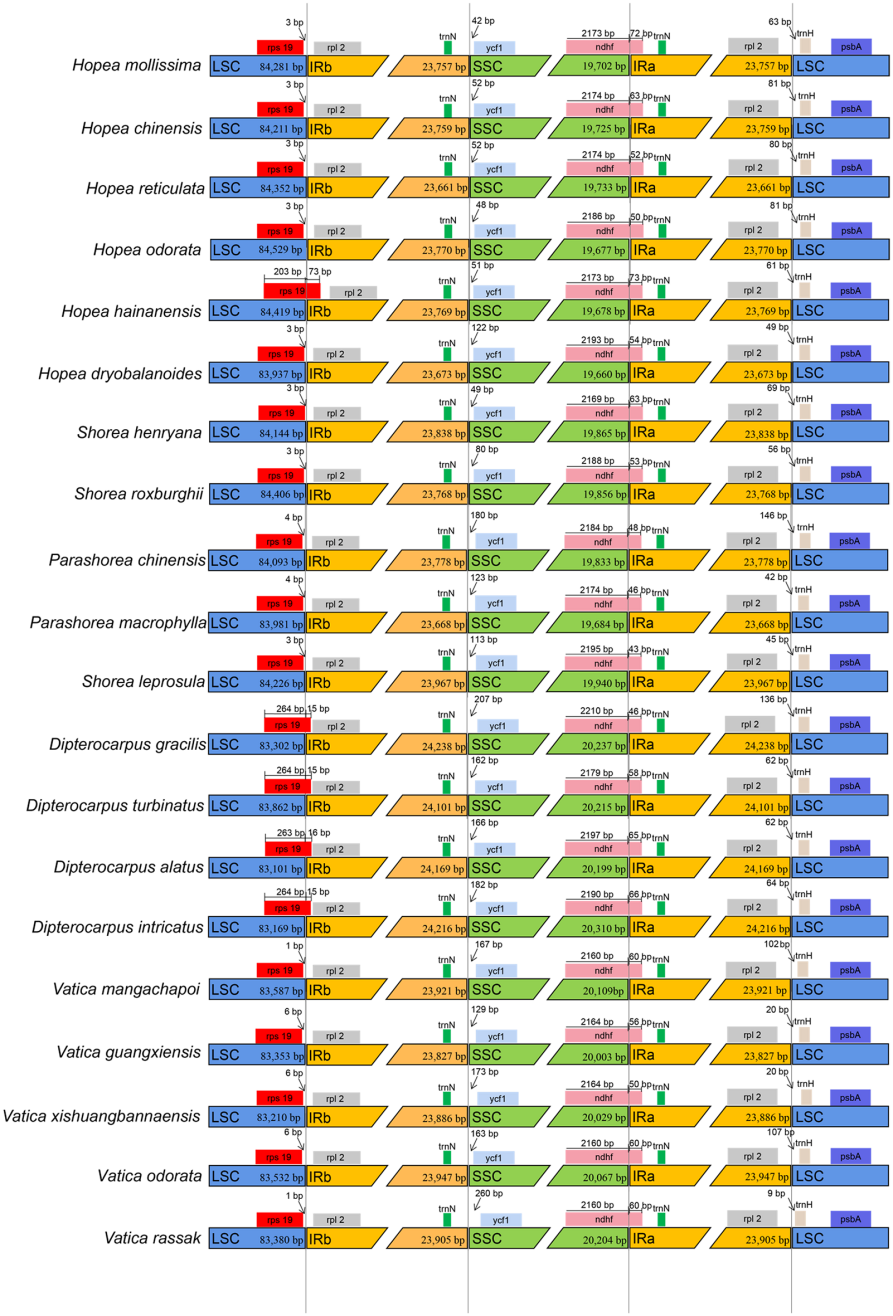
Comparison of the borders of the all regions among 20 chloroplast genomes of Dipterocarpoideae

The contraction and expansion of IR regions are the main contributors to the size variation in cp genomes and alter the evolutionary rate of the cp genome [41, 42]. We compared the IR boundaries in 20 Dipterocarpoideae species and found that the IR boundary regions varied slightly, especially IRb/SSC, SSC/IRa, and IRa/LSC (Fig. 4). At the junction of LSC and IRb regions, the *rps19* gene was found completely covered by the LSC region in most species but extended into IRb region in only four Dipterocarpus species and *H.hainanensis*, while *rpl2* was present completely in the IR regions. The analysis of the IRb/SSC junction showed the complete presence of *ycf1* in the SSC region. The *ndhF* was found at the junction of IRa/SSC. The size of *ndhF* in IRa was ranged from 43 to 73 bp. The IR boundary characteristics of all other species were conserved, the contraction and expansion were not obvious in Dipterocarpoideae.

### Codon usage and amino acid frequency

To characterize the evolution of the codon usage in the Dipterocarpoideae species, we measured the codon usage bias of all protein-coding genes in cp genome of the eleven species (Tables 3 and 4, Additional file 2: Table S1). We calculated the codon usage bias through the relative synonymous codon usage (RSCU). In addition to the normal ATG start codon that encodes formyl-methionine, alternative start codons have also been found in Araceae species, including ACG, ATA, and GTG [43]. However, in our research, start codon was only ATG with no amino acid bias. While the arginine (Arg), leucine (Leu) and serine (Ser) were encoded by six codons with the highest preferences. Especially, the maximum (1.73–1.98) and minimum (0.43–0.51) values of RSCU were found in Arg (except *H. odorata*). In addition, the G/C at 3′end content values were 32.5% in *H. odorata* to 37.8% in *V. odorata*, which indicates that these genes preferred the codons ended with A/U. Other indicators that related to RSCU are relatively conserved among species, including the codon adaptation index (CAI), the codon usage index (CBI), frequency of optimal codons (Fop), the effective number of codons (ENc) and GC content of synonymous third codons positions (GC3s) .

**Table 3:** The indexes of the codon usage bias of protein-coding genes of Dipterocarpoideae

	CAI	CBI	Fop	ENc	GC3s
*D.gracilis*	0.164	− 0.079	0.369	55.47	0.359
*D. alatus*	0.155	− 0.082	0.366	57.93	0.413
*D.intricatus*	0.158	− 0.105	0.363	55.08	0.369
*H.mollissima*	0.162	− 0.078	0.365	56.08	0.366
*H.odorata*	0.169	− 0.082	0.369	53.64	0.329
*S.leprosula*	0.168	− 0.087	0.365	53.99	0.332
*S.roxburghii*	0.166	− 0.085	0.366	54.1	0.335
*S.henryana*	0.161	− 0.079	0.364	56.05	0.365
*V. xishuangbannaensis*	0.157	− 0.082	0.364	57.19	0.394
*V.odorata*	0.158	− 0.096	0.362	56.41	0.378
*V. rassak*	0.163	− 0.088	0.366	56.22	0.376

**Table 4:** Codon content of 20 amino acids and stop codons in *H.odorata*

*H. odorata*							
AA	Codons	Numbers	RSCU	AA	Codons	Numbers	RSCU
Phe	UUU	1187	1.25	Ser	UCU	787	1.63
	UUC	707	0.75		UCC	486	1.01
Leu	UUA	763	1.61		UCA	545	1.13
	UUG	606	1.28		UCG	307	0.64
	CUU	635	1.34	Pro	CCU	445	1.32
	CUC	255	0.54		CCC	302	0.9
	CUA	365	0.77		CCA	377	1.12
	CUG	226	0.48		CCG	225	0.67
Ile	AUU	1126	1.44	Thr	ACU	510	1.33
	AUC	618	0.79		ACC	348	0.91
	AUA	605	0.77		ACA	441	1.15
Met	AUG	593	1		ACG	236	0.61
Val	GUU	571	1.44	Ala	GCU	606	1.61
	GUC	235	0.59		GCC	264	0.7
	GUA	520	1.31		GCA	390	1.04
	GUG	259	0.65		GCG	246	0.65
Tyr	UAU	879	1.44	Cys	UGU	329	1.17
	UAC	338	0.56		UGC	233	0.83
TER	UAA	292	0.94	TER	UGA	410	1.32
	UAG	228	0.74	Trp	UGG	550	1
His	CAU	567	1.46	Arg	CGU	365	0.97
	CAC	211	0.54		CGC	180	0.48
Gln	CAA	772	1.47		CGA	472	1.26
	CAG	281	0.53		CGG	208	0.55
Asn	AAU	1006	1.42	Ser	AGU	481	1
	AAC	410	0.58		AGC	286	0.59
Lys	AAA	1081	1.4	Arg	AGA	686	1.83
	AAG	458	0.6		AGG	339	0.9
Asp	GAU	875	1.53	Gly	GGU	603	1.13
	GAC	267	0.47		GGC	270	0.51
Glu	GAA	1086	1.39		GGA	802	1.5
	GAG	480	0.61		GGG	458	0.86

### Repeat analyses

**Fig. 5:**
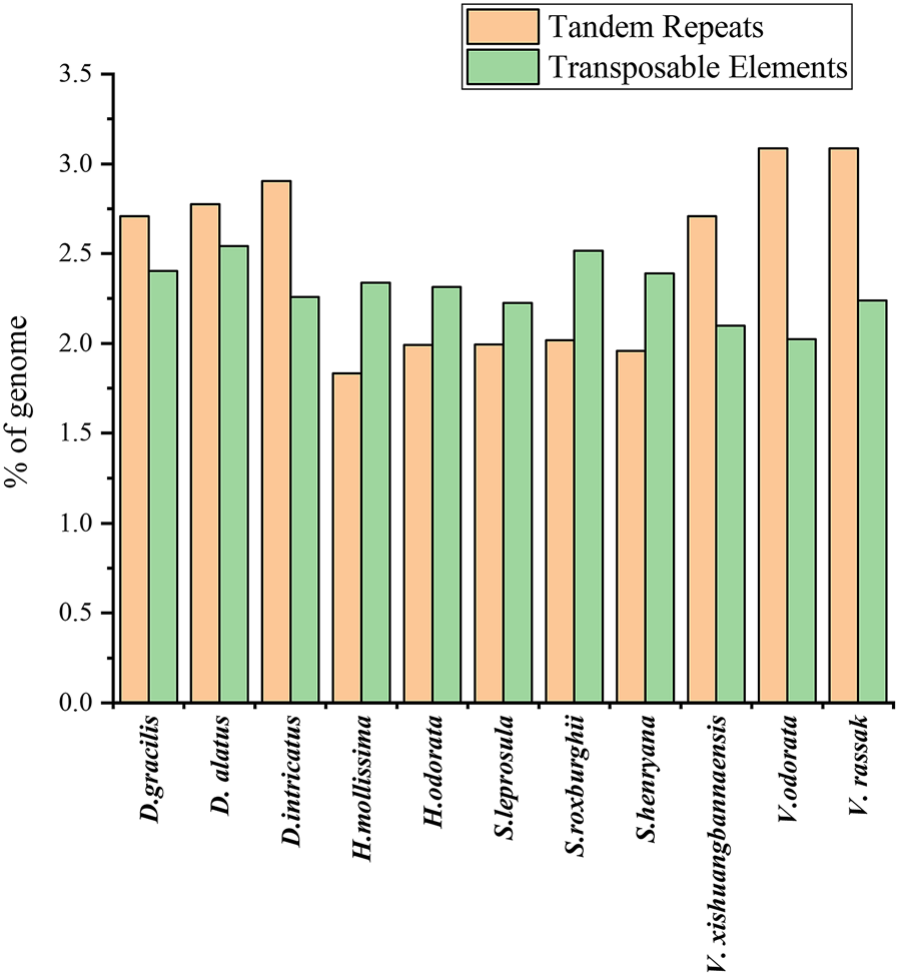
The repetitive sequence in eleven Dipterocarpoideae cp genomes used TandemRepeatFinder and RepeatMasker

We used two methods (TandemRepeatFinder and RepeatMasker) to analyze the repetitive sequence in eleven Dipterocarpoideae cp genomes (Fig. 5). The results showed that there were slightly more tandem repeats in cp genomes of Dipterocarpoideae, while the cp genomes of *Hopea* and *Shorea* retained slightly more transposable factors (TE) than the tandem repeats. The numbers of four types TEs repeats in the eleven Dipterocarpoideae cp genomes were similar and conserved (Table 5) LTR (long terminal repeats) was the most abundant TE followed by DNA and LINE (long interspersed nuclear elements).

**Table 5:** Numbers of the TE repeat types in the eleven Dipterocarpoideae cp genomes

Type length (bp)	DNA	LINE	SINE	LTR	Total
*D. gracilis*	1125	150	0	2597	3872
*D. alatus*	1122	150	0	2581	3853
*D. intricatus*	1106	50	0	2494	3650
*H. mollissima*	1021	228	0	2499	3748
*H. odorata*	1012	228	0	2479	3719
*S. leprosula*	1019	147	0	2426	3592
*S. roxburghii*	1197	147	0	2701	4045
*S. henryana*	1191	147	0	2630	3849
*V. xishuangbannaensis*	1017	90	0	2086	3170
*V. odorata*	912	51	0	2376	3316
*V. rassak*	1083	39	0	2268	3390

### Selective pressure analysis

**Fig. 6:**
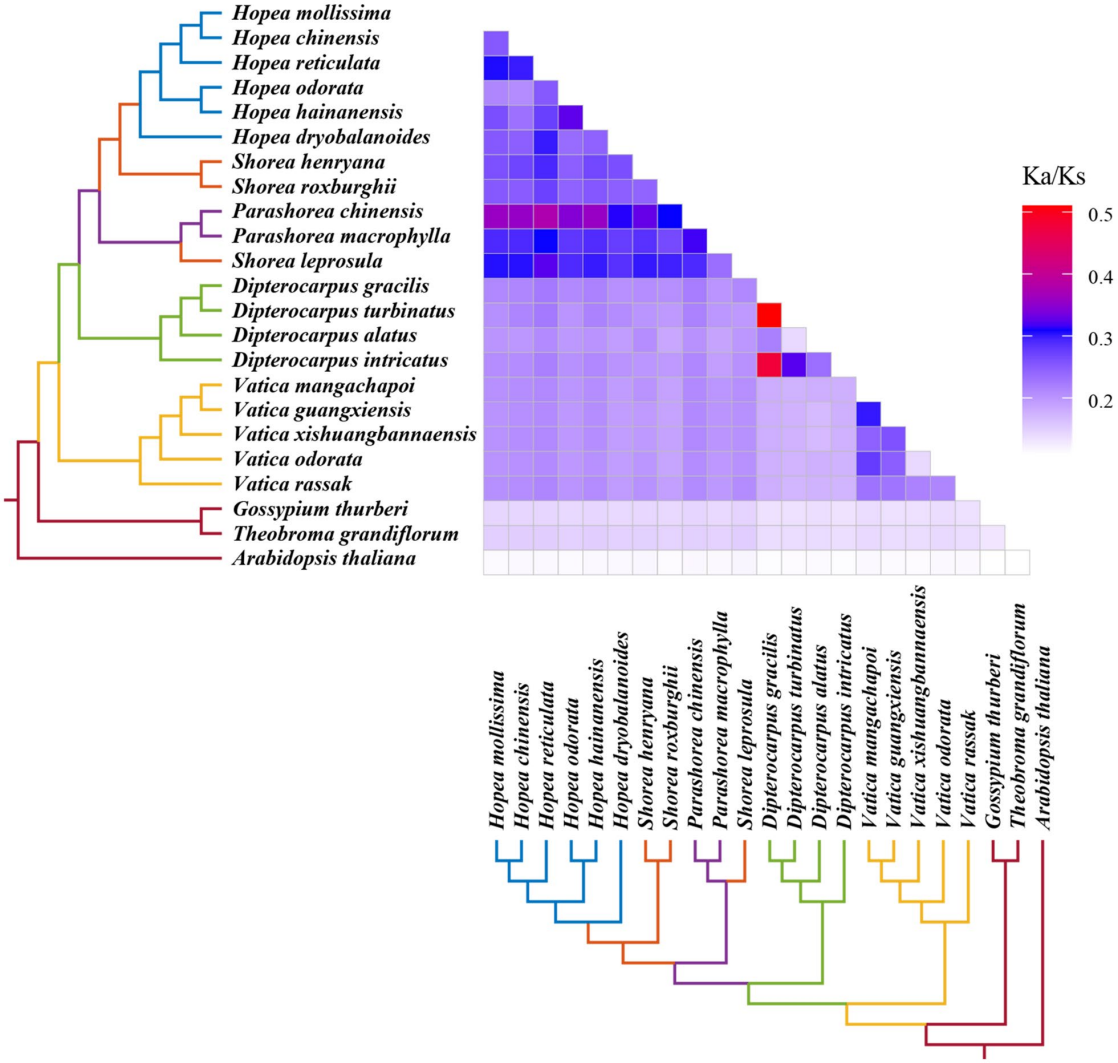
A comparison of pairwise K_a_/K_s_ values of 23 species concatenated all single copy gene sequences

The pairwise K_a_/K_s_ ratios of all 23 species pair were calculated using the concatenated 50 single-copy genes alignments (Fig. 6). The ratios among species of Dipterocarpaceae were much higher than those involving the outgroups. The K_a_/K_s_ ratios of *D. gracilis*-*D. intricatus* pair and *D. gracilis*-*D. turbinatus* pair were detected highes. The elevated K_a_/K_s_ ratios are unlikely to be explained by changes in codon preference since we did not obtain obvious codon usage bias in Dipterocarpoideae species (Additional file 2: Table S1). So we consider that it may be an indication of an elevated mutation rate that caused the K_a_/K_s_ ratios exceptionally high. We observed similar phenomenon in other research and they also inferred that high K_a_/K_s_ ratios was caused by elevated mutation rate [44].

**Fig. 7:**
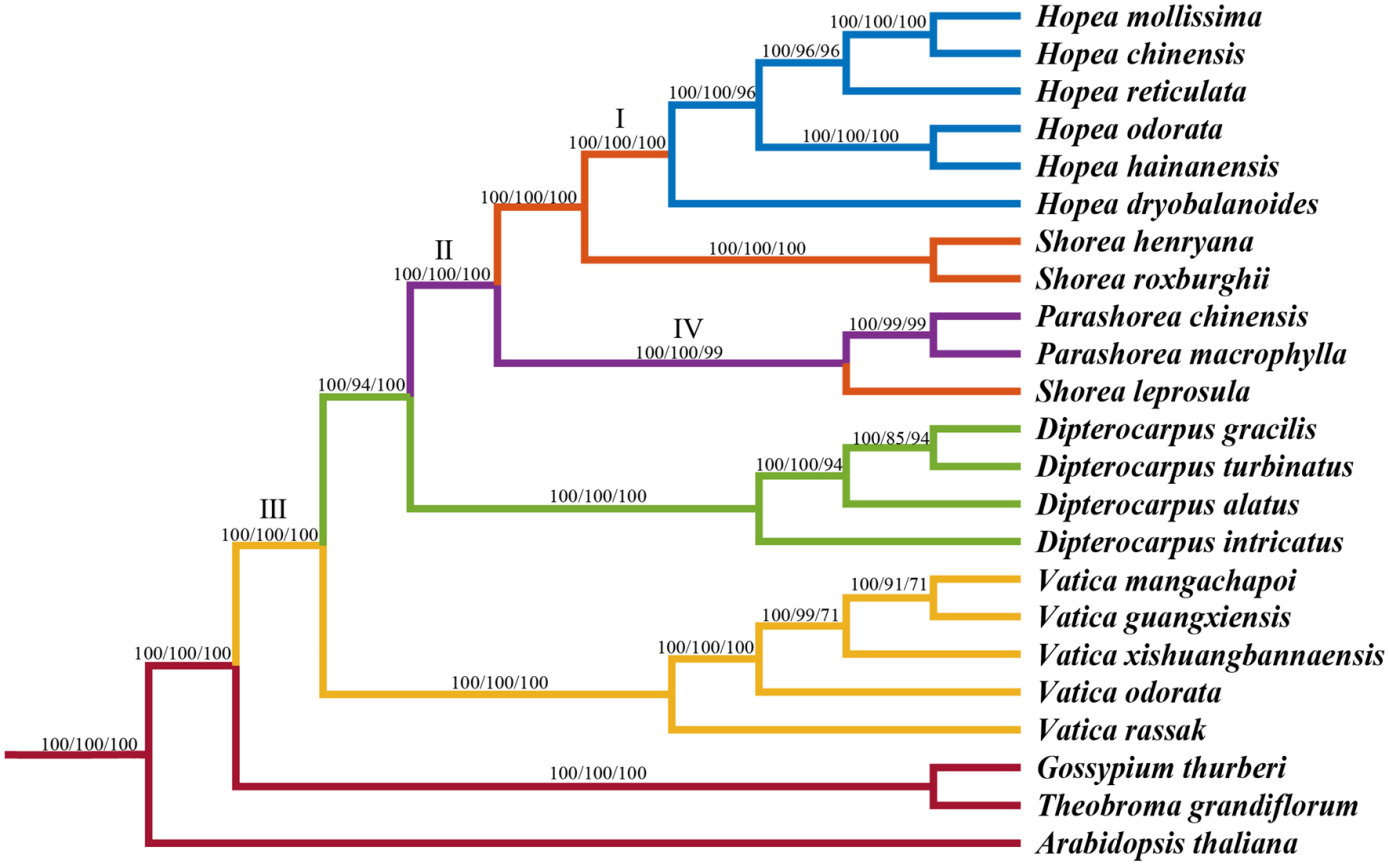
Phylogenetic relationships of genus Dipterocarpoideae species with related species based on 50 single-copy genes. The topology is indicated with BI/ML/NJ bootstrap support values at each node. Roman scrip (I/II/III/IV) represent positively selected branches

**Fig. 8:**
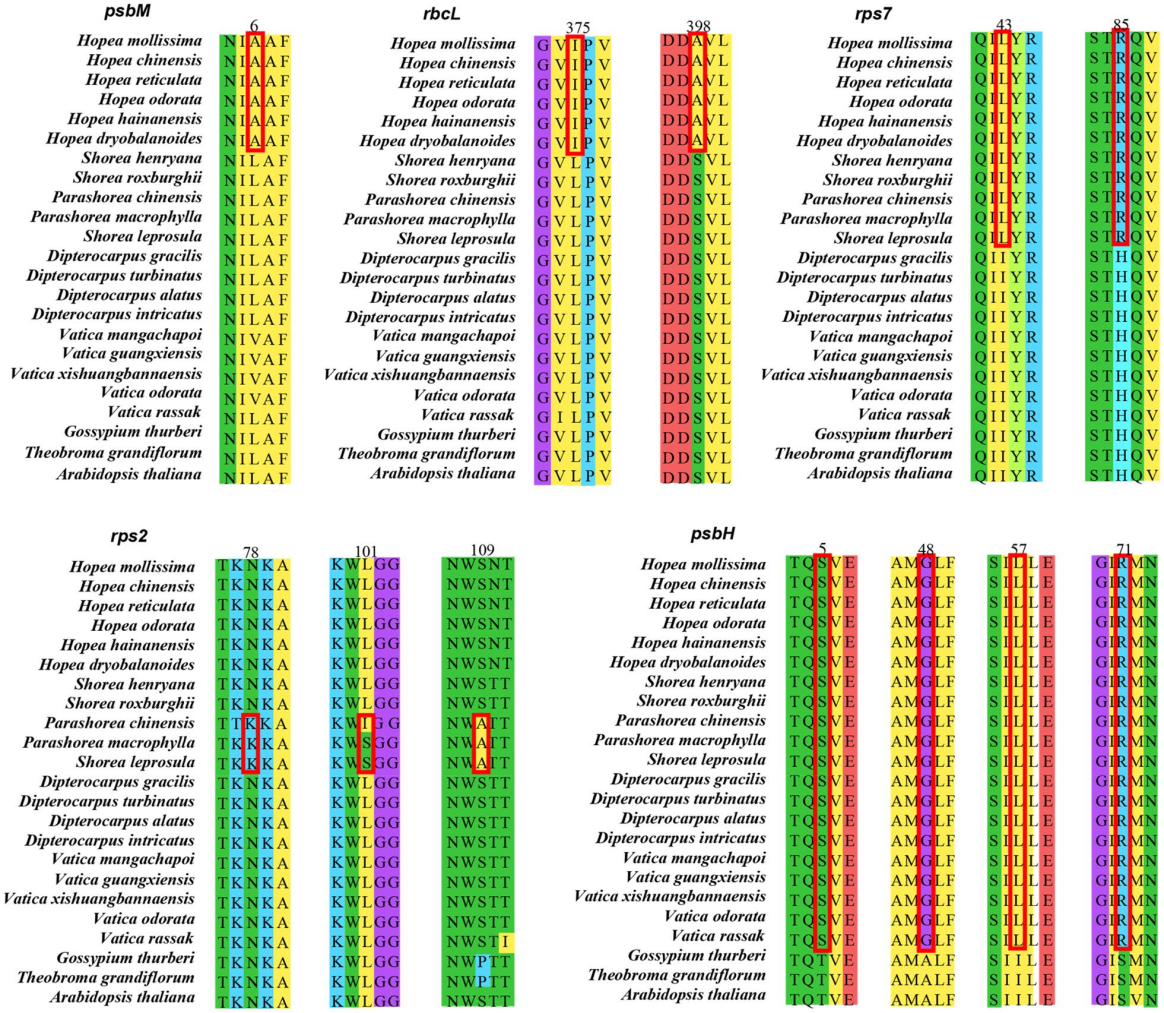
Comparison of partial site under positive selection of different genes

Since the short episodes of positive selection signal at of some sites may be masked by the long-term history of purification selection in the paired K_a_/K_s_ test, we carried out positive selection test using the branch-site model implemented in PAML. The results showed that five genes (*psbM, rbcL, rps7, rps2, psbH*) have been positively selected (*p* < 0.05) at four branches (Figs. 7 and 8; Table 6). Among them, four genes had more than one positively selected site. The *psbH* gene at branch III which was ancestor of 20 Dipterocarpoideae species, with four positively selected sites, *rps2* gene at branch IV possessed three sites under positive selection, followed by *rps7* and *rbcL* at branch II and I had two positively selected sites, *psbM* gene at branch I possessed one positively selected site. The *psbH* gene was subjected to positive selection in the common ancestor of all the 20 species of Dipterocarpoideae (T5S, A48G, I57L, S71R) compared with three outgroups. And when we observed the alignment matrix of PSBM encoded by *psbM*, we found that the sixth amino acid was Alanine (A) in all *Hopea* species, but was Leucine (L) or Valine (V) in other species. In addition, the six *Hopea* species (*H. mollissima*, *H. chinensis*, *H. reticulata*, *H. odorata*, *H. hainanensis*, *H. dryobalanoides*) have specific mutations at two positions in *rbcL* gene (I375L, A398S).

**Table 6:** Test of positively selected sites in species based on branch-site model

Genename	Branch	*p*-value	BEB
*psbM*	I	0.014	6:L:0.987*
*rbcL*	I	0.047	375:L:0.765;398:S:0.655
*rps7*	II	0.011	43:I:0.684;85:H:0.839
*psbH*	III	0.004	5:T:0.807;48:A:0.752;57:I:0.754;71:S:0.974*
*rps2*	IV	0.002	78:N:0.926;101:L:0.786;109:S:0.648

* The sites with a posterior probability of positive selection over 0.95

### Phylogenetic relationships among Dipterocarpoideae

A total of 20 Dipterocarpoideae cp genomes were used to perform phylogenetic analysis. *Gossypium thurberi*, *Theobroma grandiflorum* and *Arabidopsis thaliana* were used as the outgroups. The phylogenetic tree was constructed using Bayesian analysis (BI), ML and NJ methods based on 50 single-copy genes (Fig. 7). All phylogenetic trees have the same topology. The bootstrap values of almost nodes were equal to 100. Each genus clustered together to form a single clade except *Shorea* in which most species clustered together while *Shorea leprosula* clustered with the *Parashorea* species which has been reported by Jacqueline Heckenhauer et al. [45].

### Analysis of chloroplast barcoding loci

DNA barcoding is currently an effective and widely used tool that enables rapid and accurate identification of plant species. We found a number of potential marker genes (*accD, matK, rbcL, rpoA, rpoB, rpoC1, rpoC2, ycf1* and *ndhF*) [46, 47] that may be used in identification of Dipterocarpoideae. Then, two criteria were satisfied for an ideal candidate DNA barcoding locus: (i) Sequences in all 20 species are divergent (ii) The phylogenetic trees based on the marker gene through the ML method with the same parameters are almost the same as the tree based on single-copy genes [46, 48]. The average *p*-distance values between 20 species of *rpoC2* were 0.014-0.021 (Additional file 3: Table S2) which were larger than the average value in protein-coding genes of Magnoliaceae [49]. After filtering with these two criteria, only the *rpoC2* gene was left, suggestiong that *rpoC2* was a potential cp barcoding locus of Dipterocarpoideae (Additional file 3: Table S2).

## Discussion

Our comparison of cp genome structure and content of all the 20 cp genomes in the same family showed that the gene content and genome organization are conserved across species in this family. There were some differences of *rps16* and *ycf15* among the species. In our eleven cp genomes, only three species lack *rps16* (*D. gracilis*, *D. alatus* and *D. intricatus*) and *ycf15* is absent in two species (*S. henryana* and *V. xishuangbannaensis*). The absence and pseudogene of the two genes have been also reported in other species [50, 51].

Codon usage changes have important contribution to cp genome evolution [52], and our results showed that codon usage bias was conserved across species in Dipterocarpoideae. In addition, most codons preferentially ended with A/U with RSCU≥1, suggesting that certain degenerate codon usage bias was a result of the adaptive evolution of the cp genome [43]. Besides, all ENc values are larger than 53.64 and CAI, CBI and Fop value are much less than one, indicating that the codon usage biases in all the eleven species are very low.

PAML results showed low rates of evolution for all protein-coding genes in the chloroplast genomes. Five genes (*psbM, rbcL, rps7, rps2, psbH*) at four branches were under positive selection which might be due to different types of stresses faced by these species, and all the positively selected sites are in the known domains of the proteins (except the T5S and S71R sites of *psbH*). Three of the five genes, *psbM*, *rbcL* and *psbH*, are involved in photosynthesis. Those three genes may function in the growth of all Dipterocarpoideae species in adaptation to a strongly illuminated environment [53].

The phylogenetic placement of *Shorea* is not clear. D. Gamage et al. [6] and S. Indrioko et al. [54] have built the phylogenetic tree used *trnL*-*trnF* spacer, *trnL* intron, *matK* regions marker genes and *rbcL*, *petB*, *psbA*, *psaA*, and *trnL*-*trnF* regions marker genes, respectively, to build the phylogenetic tree, which showed that genus *Shorea* was not a monophyletic group. This result was not exactly consistent with the traditional taxonomy based on plant morphology. Our study generated a consistent phylogeny with high confidence on all nodes with three different phylogenetic algorithms. And we confirmed the result that *Shorea* was not monophyletic group, suggesting a new taxonomy treatment is needed for this genus.

Identification of specific plant species is helpful for the herbal medicine since the morphology of plants in the same subfamily are very similar. *D. turbinatus* has been proven with medicinal value (antibacterial, analgesic, anti-inflammatory effects and enhance anticancer efficiency) in Dipterocarpoideae analyzed in our study, so it has become necessary to develop easy and safe methods for the identification and development of Dipterocarpoideae species. In our study, *rpoC2* was a potential barcoding gene which used to be a maker to achieve accurate and rapid species identification in subfamily Dipterocarpoideae with important traditional Chinese medicine value. However, experimental verification was needed to confirm the function of the barcoding gene further.

## Conclusions

Eleven complete chloroplast genomes of Dipterocarpoideae were reported for the first time by us. Analysis of the cp genome sequences of 20 Dipterocarpoideae species showed that they had very similar cp genomic structure, gene order, codon usage and repetitive sequence features. Positive selection analysis of the genes in chloroplast genome of this subfamily showed that *psbH*, *psbM* and *rbcL* may function in the growth of all Dipterocarpoideae species in adaptation to a strongly illuminated environment. Phylogenetic analysis based on all single-copy genes of chloroplast genome showed that genus *Shorea* was not a monophyletic group, suggesting a new taxon treatment is needed for this genus. In addition, we also recommended *rpoC2* gene as a potential plant DNA barcoding locus to identify Dipterocarpoideae.

## Supplementary Information

**Additional file 1: Figure S1.** Gene map of the Dipterocarpoideae chloroplast genomes.

**Additional file 2: Table S1.** Codon content of 20 amino acids and stop codons in Dipterocarpoideae.

**Additional file 3: Table S2.** Estimates of Evolutionary Divergence between Sequences.

## Data Availability

The datasets generated during the current study are available in the National Center for Biotechnology Information database (NCBI). www.ncbi.nlm.nih.gov/ [MZ160991–MZ160998, MZ379792, MZ397800–MZ397801].

## Abbreviations

TCM: Traditional Chinese medicine
cp: Chloroplast
LSC: Large single copy
SSC: Small single copy
IR: Inverted repeat
rRNA: Ribosomal RNA
tRNA: Transfer RNA
LINE: Long interspersed nuclear elements
SINE: Short interspersed nuclear elements
LTR: Long terminal repeats
CAI: The codon adaptation index
CBI: Codon bias index
Fop: Frequency of optimal codons
ENc: The effective number of codons
GC3s: GC content of synonymous third codons positions
RSCU: The relative synonymous codon usage values
TEs: Transposable elements
BEB: Bayes Empirical Bayes
