# Supplementary material for: Comparative and phylogenetic analyses of eleven complete chloroplast genomes of Dipterocarpoideae
Source: Chin Med. 2021 Nov 25;16:125. doi: 10.1186/s13020-021-00538-8 (PMC8620154; doi:10.1186/s13020-021-00538-8)
Supplement: Supplementary file 1 — Additional file 1: Figure S1. Gene map of the Dipterocarpoideae chloroplast genomes. [file 13020_2021_538_MOESM1_ESM.pdf]

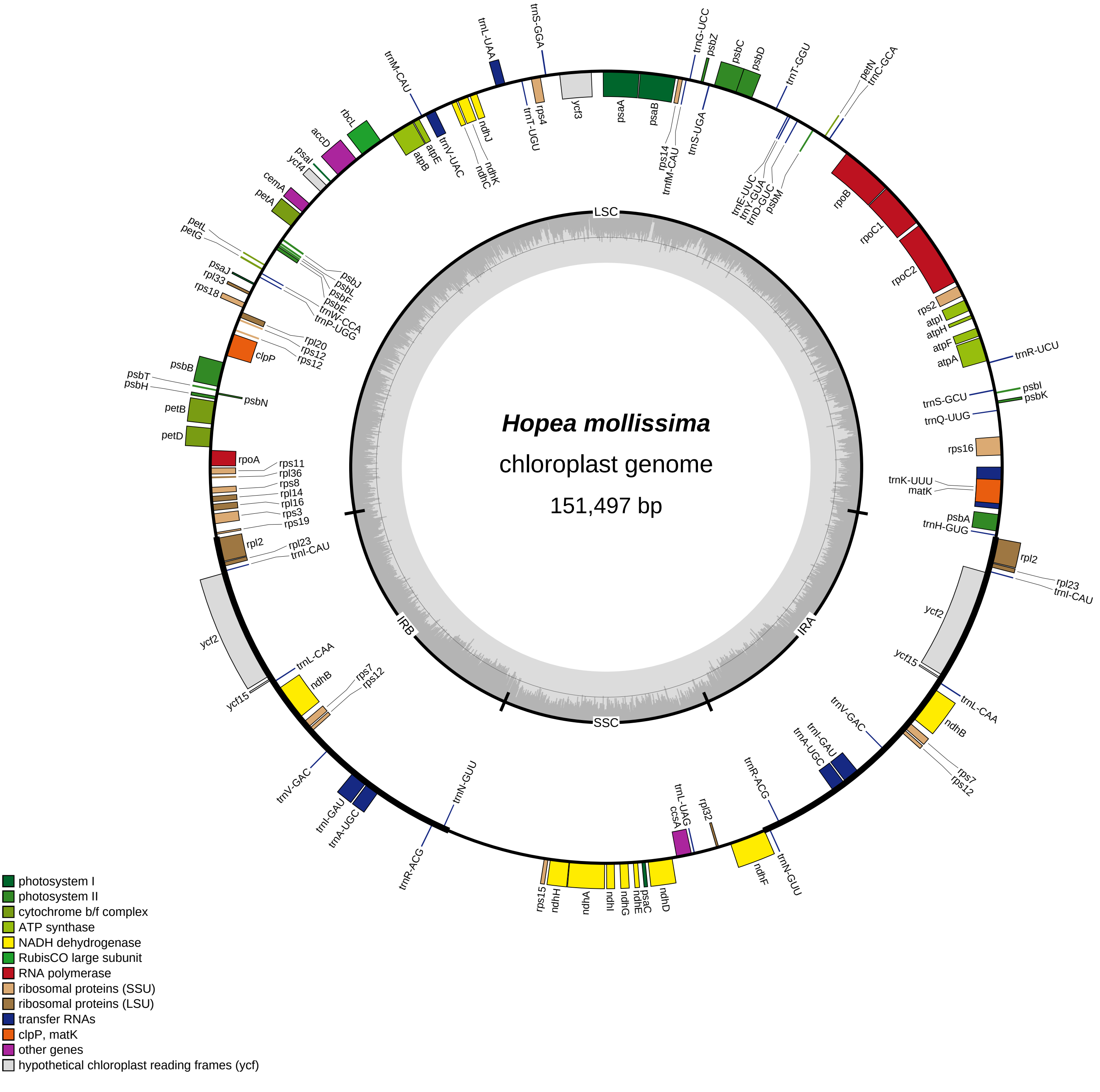

Hopea mollissima
chloroplast genome
151,497 bp
LSC
IRA
SSC
IRB
trnS-GGA
trnL-UAA
trnM-CAU
rbcL
accD
psaI
ycf4
cemA
petA
petL
petG
psaJ
rpl33
rps18
rpl20
rps12
rps12
clpP
psbB
psbT
psbH
psbN
petB
petD
rpoA
rps11
rpl36
rps8
rpl14
rpl16
rps3
rps19
rpl2
rpl23
trnI-CAU
ycf2
ycf15
trnL-CAA
ndhB
rps7
rps12
trnV-GAC
trnI-GAU
trnA-UGC
trnR-ACG
trnN-GUU
rps15
ndhH
ndhA
ndhI
ndhG
ndhE
psaC
ndhD
ccsA
trnL-UAG
rpl32
ndhF
trnN-GUU
trnR-ACG
trnA-UGC
trnI-GAU
trnV-GAC
rps12
rps7
ndhB
trnL-CAA
ycf15
ycf2
trnI-CAU
rpl23
rpl2
trnH-GUG
psbA
matK
trnK-UUU
rps16
trnQ-UUG
trnS-GCU
psbK
psbI
trnR-UCU
atpA
atpF
atpH
atpI
rps2
rpoC2
rpoC1
rpoB
trnC-GCA
petN
psbM
trnD-GUC
trnY-GUA
trnE-UUC
trnT-GGU
psbD
psbC
psbZ
trnG-UCC
trnS-UGA
trnfM-CAU
rps14
psaB
psaA
ycf3
rps4
trnT-UGU
ndhJ
ndhK
ndhC
trnV-UAC
atpE
atpB
psbJ
psbL
psbF
psbE
trnW-CCA
trnP-UGG
photosystem I
photosystem II
cytochrome b/f complex
ATP synthase
NADH dehydrogenase
RubisCO large subunit
RNA polymerase
ribosomal proteins (SSU)
ribosomal proteins (LSU)
transfer RNAs
clpP, matK
other genes
hypothetical chloroplast reading frames (ycf)

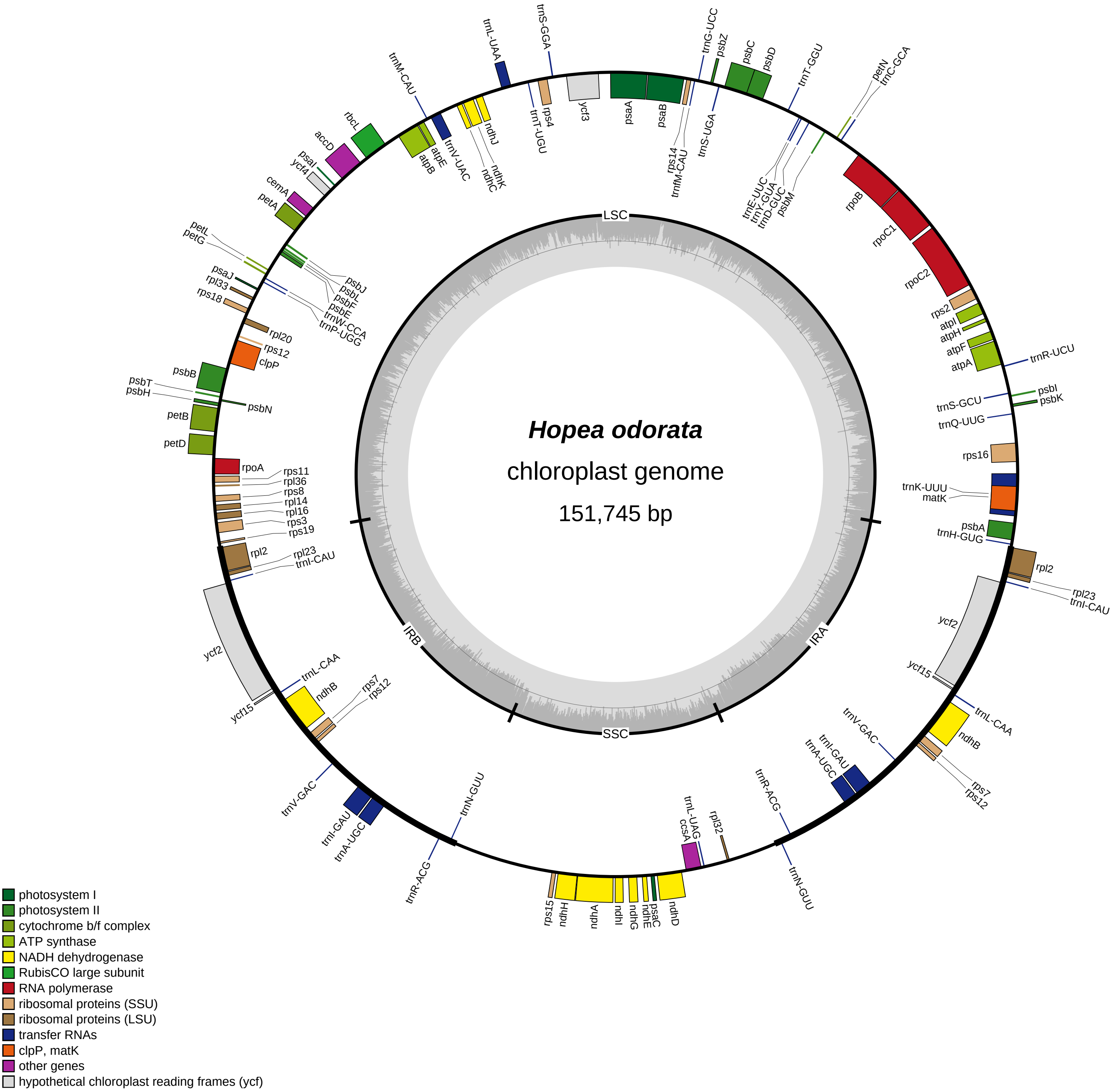
Hopea odorata
chloroplast genome
151,745 bp
LSC
IRA
SSC
IRB
trnL-UAA
trnS-GGA
trnM-CAU
trnG-UCC
psbZ
psbC
psbD
trnT-GGU
petN
trnC-GCA
rbcL
accD
psaI
ycf4
cemA
petA
atpE
atpB
trnV-UAC
ndhJ
ndhK
ndhC
trnT-UGU
rps4
ycf3
psaA
psaB
rps14
trnfM-CAU
trnS-UGA
trnE-UUC
trnY-GUA
trnD-GUC
psbM
rpoB
rpoC1
rpoC2
rps2
atpI
atpH
atpF
atpA
trnR-UCU
psbI
psbK
trnS-GCU
trnQ-UUG
rps16
trnK-UUU
matK
psbA
trnH-GUG
rpl2
rpl23
trnI-CAU
ycf2
ycf15
trnL-CAA
ndhB
rps7
rps12
trnV-GAC
trnI-GAU
trnA-UGC
trnR-ACG
trnN-GUU
trnL-UAG
ccsA
rpl32
rps15
ndhH
ndhA
ndhI
ndhG
ndhE
psaC
ndhD
petL
petG
psbJ
psbL
psbF
psbE
trnW-CCA
trnP-UGG
psaJ
rpl33
rps18
rpl20
rps12
clpP
psbB
psbT
psbH
psbN
petB
petD
rpoA
rps11
rpl36
rps8
rpl14
rpl16
rps3
rps19
photosystem I
photosystem II
cytochrome b/f complex
ATP synthase
NADH dehydrogenase
RubisCO large subunit
RNA polymerase
ribosomal proteins (SSU)
ribosomal proteins (LSU)
transfer RNAs
clpP, matK
other genes
hypothetical chloroplast reading frames (ycf)

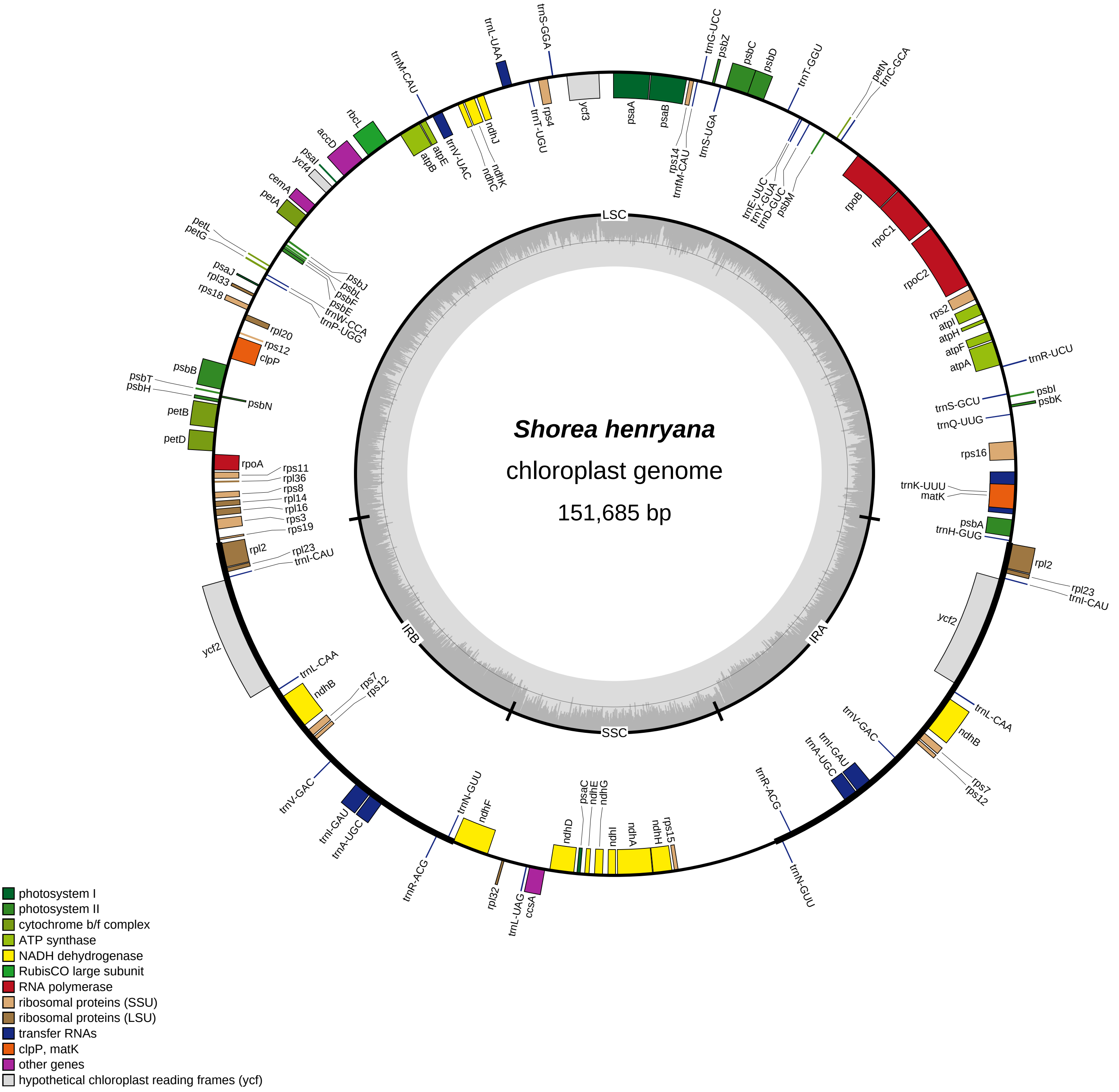
Shorea henryana
chloroplast genome
151,685 bp
LSC
IRA
SSC
IRB
trnL-UAA
trnS-GGA
trnM-CAU
rbcL
accD
psaI
ycf4
cemA
petA
petL
petG
psaJ
rpl33
rps18
rpl20
rps12
clpP
psbB
psbT
psbH
psbN
petB
petD
rpoA
rps11
rpl36
rps8
rpl14
rpl16
rps3
rps19
rpl2
rpl23
trnI-CAU
ycf2
trnL-CAA
ndhB
rps7
trnV-GAC
trnI-GAU
trnA-UGC
trnR-ACG
trnN-GUU
ndhF
rpl32
trnL-UAG
ccsA
ndhD
psaC
ndhE
ndhG
ndhI
ndhA
ndhH
rps15
psbJ
psbL
psbF
psbE
trnW-CCA
trnP-UGG
atpB
atpE
trnV-UAC
ndhC
ndhK
ndhJ
rps4
trnT-UGU
ycf3
psaA
psaB
rps14
trnfM-CAU
trnS-UGA
trnG-UCC
psbZ
psbC
psbD
trnT-GGU
trnE-UUC
trnY-GUA
trnD-GUC
psbM
petN
trnC-GCA
rpoB
rpoC1
rpoC2
rps2
atpI
atpH
atpF
atpA
trnR-UCU
trnS-GCU
trnQ-UUG
psbI
psbK
rps16
trnK-UUU
matK
psbA
trnH-GUG
photosystem I
photosystem II
cytochrome b/f complex
ATP synthase
NADH dehydrogenase
RubisCO large subunit
RNA polymerase
ribosomal proteins (SSU)
ribosomal proteins (LSU)
transfer RNAs
clpP, matK
other genes
hypothetical chloroplast reading frames (ycf)

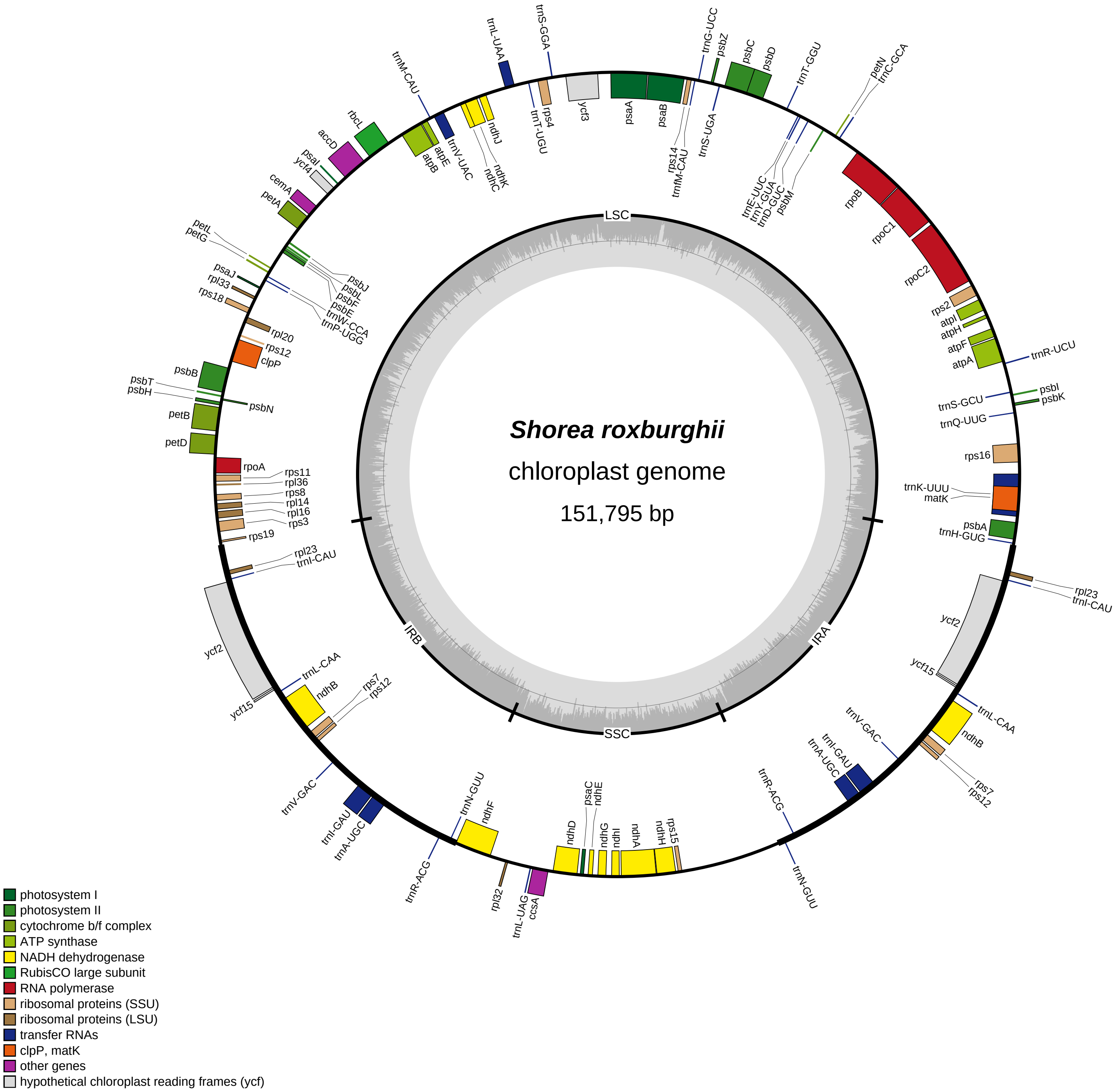
Shorea roxburghii
chloroplast genome
151,795 bp
LSC
IRA
SSC
IRB
trnS-GGA
trnL-UAA
trnM-CAU
rbcL
accD
psaI
ycf4
cemA
petA
petL
petG
psaJ
rpl33
rps18
rpl20
rps12
clpP
psbB
psbT
psbH
psbN
petB
petD
rpoA
rps11
rpl36
rps8
rpl14
rpl16
rps3
rps19
rpl23
trnI-CAU
ycf2
ycf15
trnL-CAA
ndhB
rps7
rps12
trnV-GAC
trnI-GAU
trnA-UGC
trnR-ACG
trnN-GUU
ndhF
rpl32
trnL-UAG
ccsA
ndhD
psaC
ndhE
ndhG
ndhI
ndhA
ndhH
rps15
trnN-GUU
trnR-ACG
trnA-UGC
trnI-GAU
trnV-GAC
rps12
rps7
ndhB
trnL-CAA
ycf15
ycf2
trnI-CAU
rpl23
trnH-GUG
psbA
matK
trnK-UUU
rps16
trnQ-UUG
trnS-GCU
psbK
psbI
trnR-UCU
atpA
atpF
atpH
atpI
rps2
rpoC2
rpoC1
rpoB
trnC-GCA
petN
psbM
trnD-GUC
trnY-GUA
trnE-UUC
trnT-GGU
psbD
psbC
psbZ
trnG-UCC
trnS-UGA
trnfM-CAU
rps14
psaB
psaA
ycf3
rps4
trnT-UGU
ndhJ
ndhK
ndhC
trnV-UAC
atpE
atpB
psbJ
psbL
psbF
psbE
trnW-CCA
trnP-UGG
photosystem I
photosystem II
cytochrome b/f complex
ATP synthase
NADH dehydrogenase
RubisCO large subunit
RNA polymerase
ribosomal proteins (SSU)
ribosomal proteins (LSU)
transfer RNAs
clpP, matK
other genes
hypothetical chloroplast reading frames (ycf)

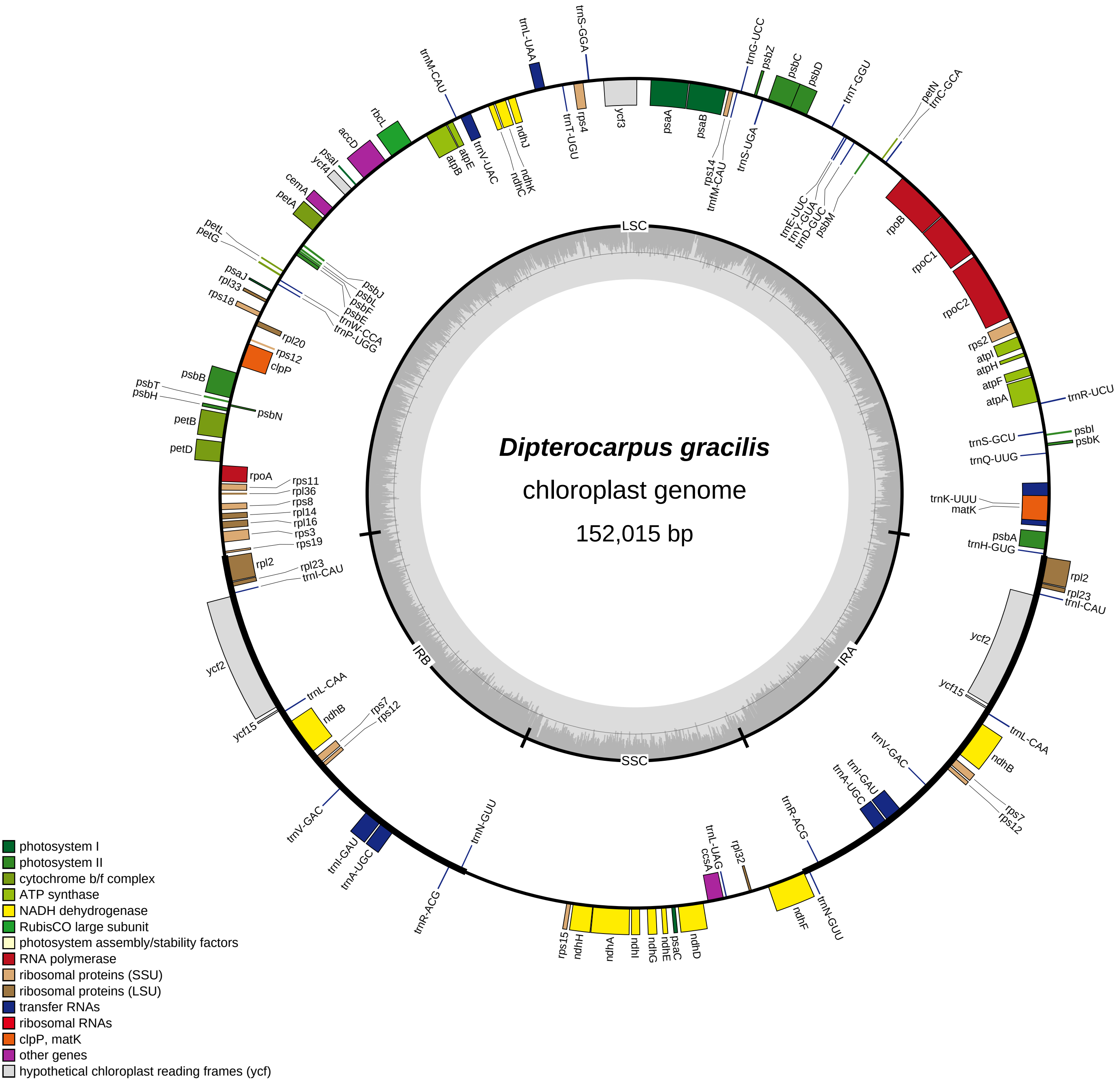
Dipterocarpus gracilis
chloroplast genome
152,015 bp
LSC
IRA
SSC
IRB
trnS-GGA
trnL-UAA
trnM-CAU
trnT-UGU
rps4
ycf3
psaA
psaB
rps14
trnfM-CAU
trnG-UCC
psbZ
trnS-UGA
psbC
psbD
trnT-GGU
trnE-UUC
trnY-GUA
trnD-GUC
psbM
petN
trnC-GCA
rpoB
rpoC1
rpoC2
rps2
atpI
atpH
atpF
atpA
trnR-UCU
psbI
psbK
trnS-GCU
trnQ-UUG
trnK-UUU
matK
psbA
trnH-GUG
rpl2
rpl23
trnI-CAU
ycf2
ycf15
trnL-CAA
ndhB
rps7
rps12
trnV-GAC
trnI-GAU
trnA-UGC
trnR-ACG
trnN-GUU
ndhF
rpl32
trnL-UAG
ccsA
ndhD
psaC
ndhE
ndhG
ndhI
ndhA
ndhH
rps15
trnN-GUU
trnR-ACG
trnA-UGC
trnI-GAU
trnV-GAC
rps12
rps7
ndhB
trnL-CAA
ycf15
ycf2
trnI-CAU
rpl23
rpl2
rps19
rps3
rpl16
rpl14
rps8
rpl36
rps11
rpoA
petD
petB
psbN
psbH
psbT
psbB
clpP
rps12
rpl20
rps18
rpl33
psaJ
trnP-UGG
trnW-CCA
psbE
psbF
psbL
psbJ
petG
petL
petA
cemA
ycf4
psaI
accD
rbcL
atpB
atpE
trnV-UAC
ndhC
ndhK
ndhJ
photosystem I
photosystem II
cytochrome b/f complex
ATP synthase
NADH dehydrogenase
RubisCO large subunit
photosystem assembly/stability factors
RNA polymerase
ribosomal proteins (SSU)
ribosomal proteins (LSU)
transfer RNAs
ribosomal RNAs
clpP, matK
other genes
hypothetical chloroplast reading frames (ycf)

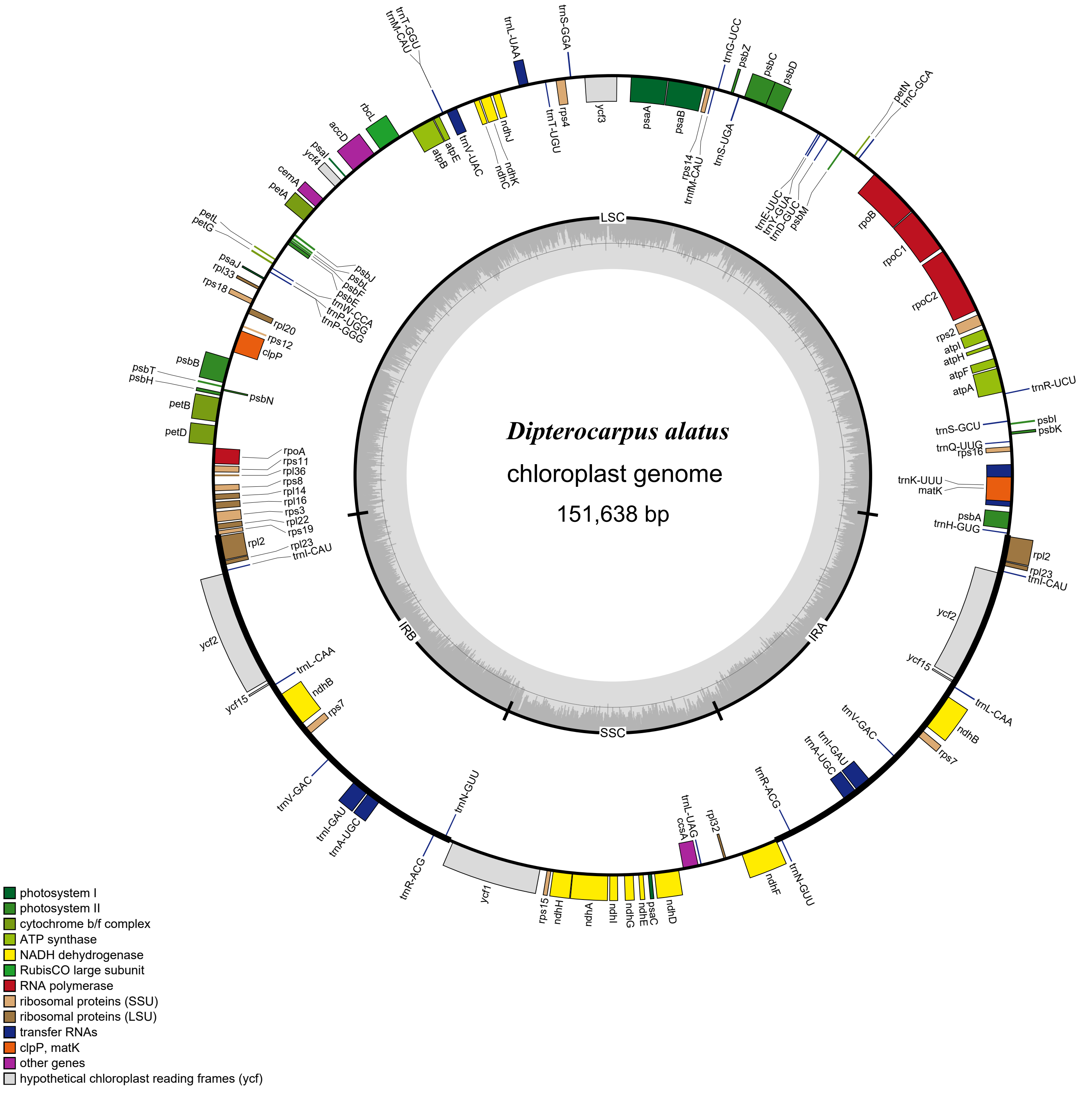

Dipterocarpus alatus
chloroplast genome
151,638 bp
LSC
IRA
SSC
IRB
trnT-GGU
trnM-CAU
trnL-UAA
trnS-GGA
trnG-UCC
psbZ
psbC
psbD
petN
trnC-GCA
rbcL
accD
psaI
ycf4
cemA
petA
atpE
atpB
trnV-UAC
ndhJ
ndhK
ndhC
trnT-UGU
rps4
ycf3
psaA
psaB
rps14
trnfM-CAU
trnS-UGA
trnE-UUC
trnY-GUA
trnD-GUC
psbM
rpoB
rpoC1
rpoC2
rps2
atpI
atpH
atpF
atpA
trnR-UCU
trnS-GCU
psbI
psbK
trnQ-UUG
rps16
trnK-UUU
matK
psbA
trnH-GUG
rpl2
rpl23
trnI-CAU
ycf2
ycf15
trnL-CAA
ndhB
rps7
trnV-GAC
trnI-GAU
trnA-UGC
trnR-ACG
trnN-GUU
ndhF
rpl32
trnL-UAG
ccsA
ndhD
psaC
ndhE
ndhG
ndhI
ndhA
ndhH
rps15
ycf1
petL
petG
psaJ
rpl33
rps18
psbJ
psbL
psbF
psbE
trnW-CCA
trnP-UGG
trnP-GGG
rpl20
rps12
clpP
psbB
psbT
psbH
psbN
petB
petD
rpoA
rps11
rpl36
rps8
rpl14
rpl16
rps3
rpl22
rps19
photosystem I
photosystem II
cytochrome b/f complex
ATP synthase
NADH dehydrogenase
RubisCO large subunit
RNA polymerase
ribosomal proteins (SSU)
ribosomal proteins (LSU)
transfer RNAs
clpP, matK
other genes
hypothetical chloroplast reading frames (ycf)

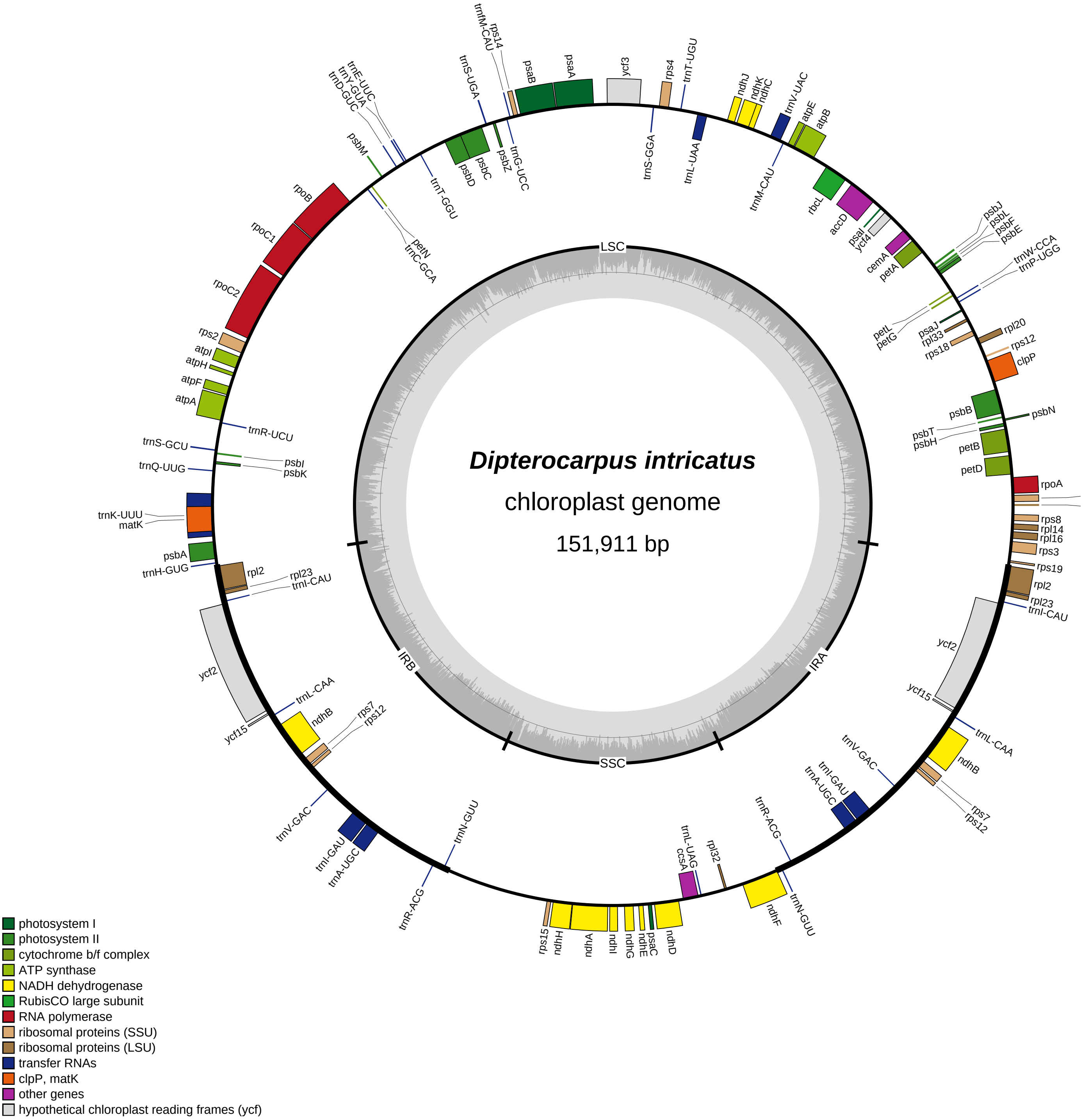

Dipterocarpus intricatus
chloroplast genome
151,911 bp
LSC
SSC
IRA
IRB
psaB
psaA
ycf3
rps4
trnT-UGU
ndhJ
ndhK
ndhC
trnV-UAC
atpE
atpB
trnfM-CAU
rps14
trnS-UGA
trnE-UUC
trnY-GUA
trnD-GUC
psbM
rpoB
rpoC1
rpoC2
rps2
atpI
atpH
atpF
atpA
trnR-UCU
trnS-GCU
trnQ-UUG
psbI
psbK
trnK-UUU
matK
psbA
trnH-GUG
rpl2
rpl23
trnI-CAU
ycf2
ycf15
trnL-CAA
ndhB
rps7
rps12
trnV-GAC
trnI-GAU
trnA-UGC
trnR-ACG
trnN-GUU
rps15
ndhH
ndhA
ndhI
ndhG
ndhE
psaC
ndhD
trnL-UAG
ccsA
rpl32
ndhF
psbD
psbC
psbZ
trnG-UCC
trnT-GGU
petN
trnC-GCA
trnS-GGA
trnL-UAA
trnM-CAU
rbcL
accD
psaI
ycf4
cemA
petA
psbJ
psbL
psbF
psbE
trnW-CCA
trnP-UGG
petL
petG
psaJ
rpl33
rps18
rpl20
clpP
psbB
psbN
psbT
psbH
petB
petD
rpoA
rps11
rpl36
rps8
rpl14
rpl16
rps3
rps19
photosystem I
photosystem II
cytochrome b/f complex
ATP synthase
NADH dehydrogenase
RubisCO large subunit
RNA polymerase
ribosomal proteins (SSU)
ribosomal proteins (LSU)
transfer RNAs
clpP, matK
other genes
hypothetical chloroplast reading frames (ycf)

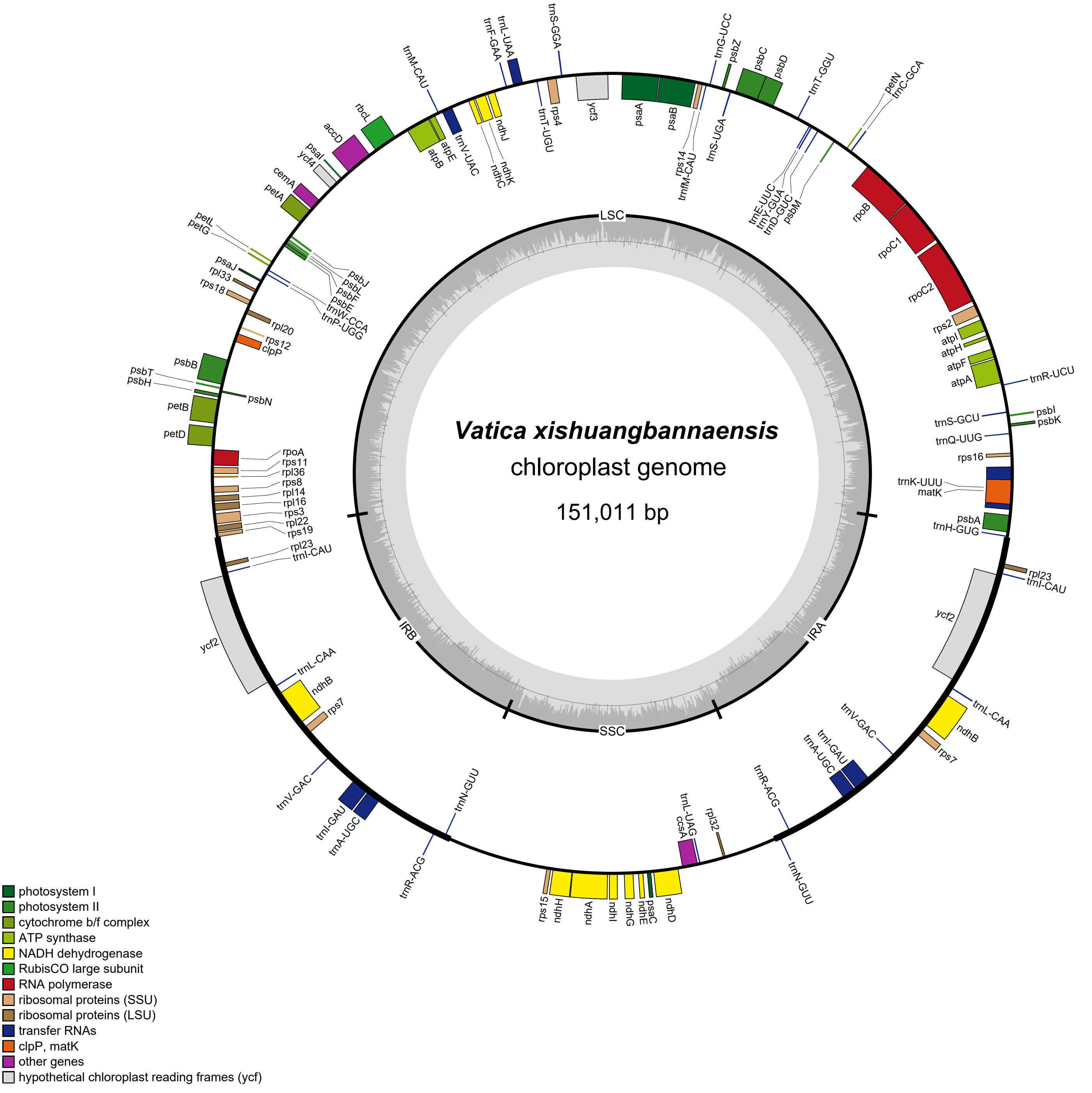

Vatica xishuangbannaensis
chloroplast genome
151,011 bp
LSC
IRA
SSC
IRB
psaA
psaB
rps14
trnfM-CAU
trnG-UCC
psbZ
trnS-UGA
psbC
psbD
trnT-GGU
trnE-UUC
trnY-GUA
trnD-GUC
psbM
petN
trnC-GCA
rpoB
rpoC1
rpoC2
rps2
atpI
atpH
atpF
atpA
trnR-UCU
psbI
psbK
trnS-GCU
trnQ-UUG
rps16
trnK-UUU
matK
psbA
trnH-GUG
rpl23
trnI-CAU
ycf2
trnL-CAA
ndhB
rps7
trnV-GAC
trnI-GAU
trnA-UGC
trnR-ACG
trnN-GUU
rpl32
trnL-UAG
ccsA
ndhD
psaC
ndhE
ndhG
ndhI
ndhA
ndhH
rps15
rpoA
rps11
rpl36
rps8
rpl14
rpl16
rps3
rpl22
rps19
petD
petB
psbH
psbT
psbN
psbB
clpP
rps12
rpl20
rps18
rpl33
psaJ
trnP-UGG
trnW-CCA
psbE
psbF
psbL
psbJ
petG
petL
petA
cemA
ycf4
psaI
accD
rbcL
atpB
atpE
trnV-UAC
trnM-CAU
ndhC
ndhK
ndhJ
trnF-GAA
trnL-UAA
trnT-UGU
rps4
trnS-GGA
ycf3
photosystem I
photosystem II
cytochrome b/f complex
ATP synthase
NADH dehydrogenase
RubisCO large subunit
RNA polymerase
ribosomal proteins (SSU)
ribosomal proteins (LSU)
transfer RNAs
clpP, matK
other genes
hypothetical chloroplast reading frames (ycf)

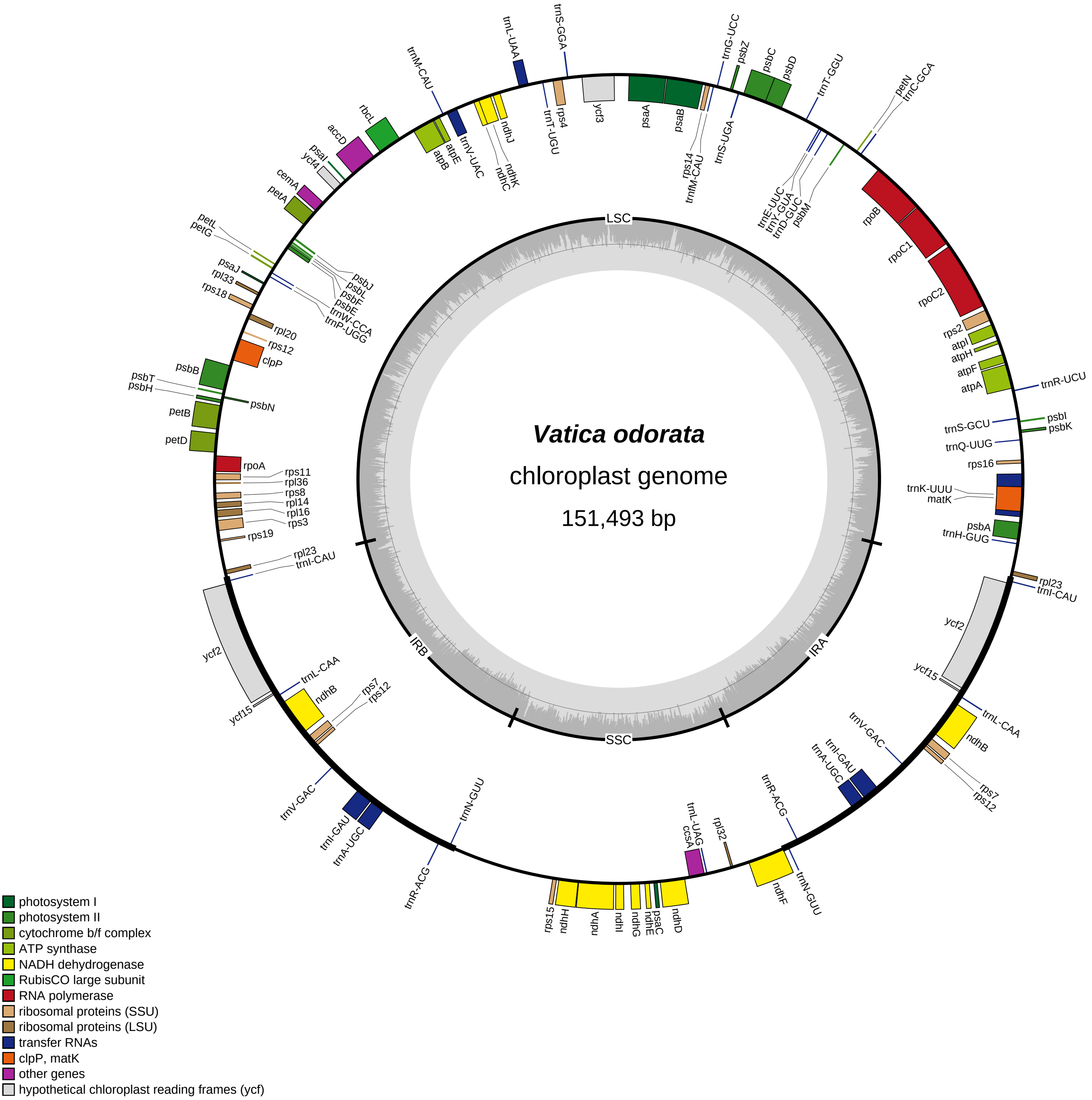

Vatica odorata
chloroplast genome
151,493 bp
LSC
SSC
IRA
IRB
trnS-GGA
trnL-UAA
trnM-CAU
rbcL
accD
psaI
ycf4
cemA
petA
petL
petG
psaJ
rpl33
rps18
rpl20
rps12
clpP
psbB
psbT
psbH
psbN
petB
petD
rpoA
rps11
rpl36
rps8
rpl14
rpl16
rps3
rps19
rpl23
trnI-CAU
ycf2
ycf15
trnL-CAA
ndhB
rps7
rps12
trnV-GAC
trnI-GAU
trnA-UGC
trnR-ACG
trnN-GUU
rps15
ndhH
ndhA
ndhI
ndhG
ndhE
psaC
ndhD
trnL-UAG
ccsA
rpl32
ndhF
trnN-GUU
trnR-ACG
trnA-UGC
trnI-GAU
trnV-GAC
rps12
rps7
ndhB
trnL-CAA
ycf15
ycf2
rpl23
trnI-CAU
trnH-GUG
psbA
matK
trnK-UUU
rps16
trnQ-UUG
trnS-GCU
psbI
psbK
trnR-UCU
atpA
atpF
atpH
atpI
rps2
rpoC2
rpoC1
rpoB
petN
trnC-GCA
trnT-GGU
psbD
psbC
psbZ
trnG-UCC
trnS-UGA
trnE-UUC
trnY-GUA
trnD-GUC
psbM
rps14
trnfM-CAU
psaB
psaA
ycf3
rps4
trnT-UGU
ndhJ
ndhK
ndhC
trnV-UAC
atpE
atpB
psbJ
psbL
psbF
psbE
trnW-CCA
trnP-UGG
photosystem I
photosystem II
cytochrome b/f complex
ATP synthase
NADH dehydrogenase
RubisCO large subunit
RNA polymerase
ribosomal proteins (SSU)
ribosomal proteins (LSU)
transfer RNAs
clpP, matK
other genes
hypothetical chloroplast reading frames (ycf)

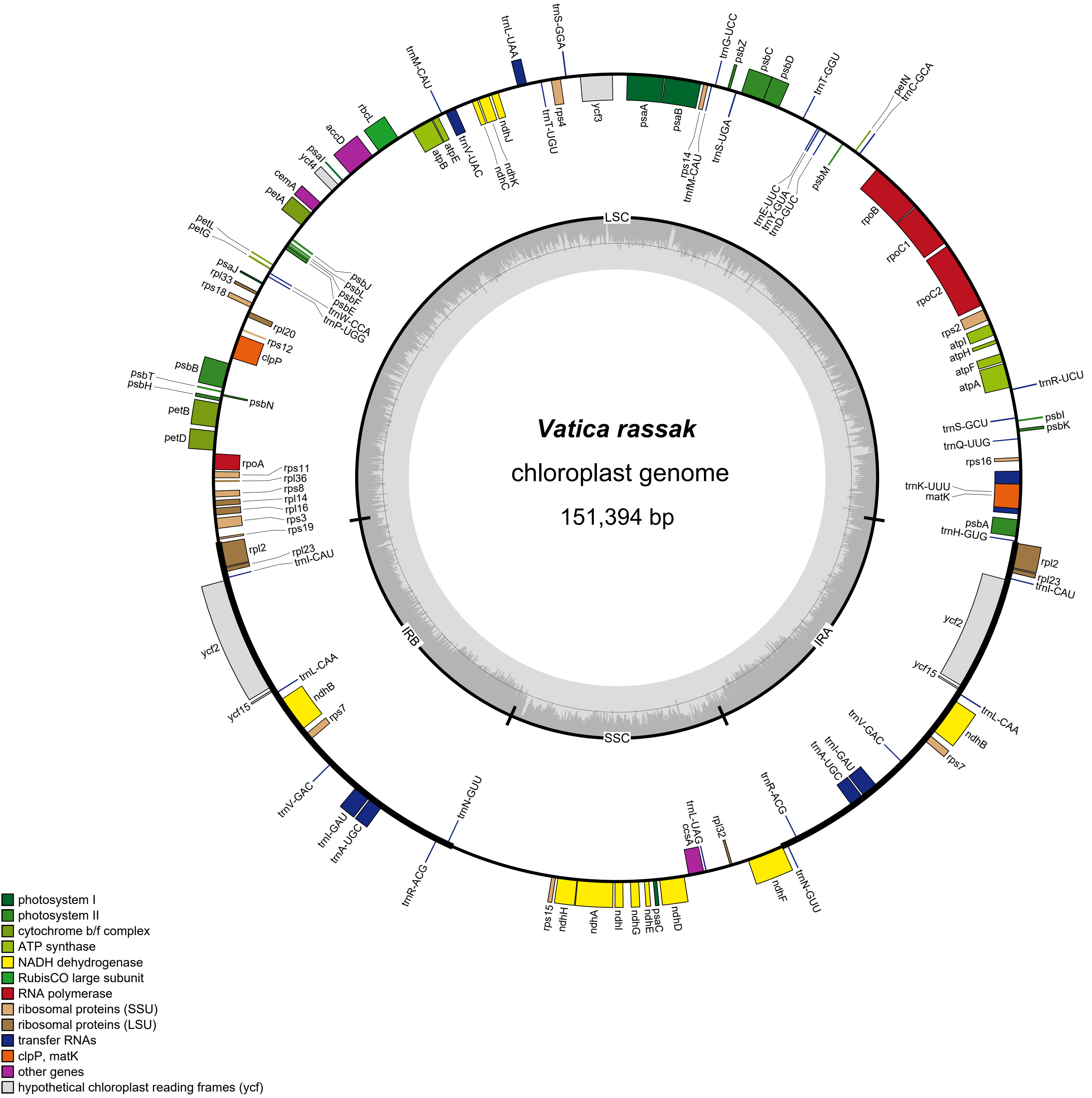

Figure S1: Gene map of the Dipterocarpoideae chloroplast genomes
